# Sidelobe reduction and capacity improvement of open-loop collaborative beamforming in wireless sensor networks

**DOI:** 10.1371/journal.pone.0175510

**Published:** 2017-05-02

**Authors:** Suhanya Jayaprakasam, Sharul Kamal Abdul Rahim, Chee Yen Leow, Tiew On Ting

**Affiliations:** 1 Wireless Systems Laboratory, Department of Electronics and Computer Engineering, Hanyang University, Seoul, South Korea; 2 Wireless Communication Centre, Universiti Teknologi Malaysia (UTM), Johor 81310, Malaysia; 3 Xi’an Jiaotong-Liverpool University, Suzhou 215133, Jiangsu Province, P.R. China; West Virginia University, UNITED STATES

## Abstract

Collaborative beamforming (CBF) with a finite number of collaborating nodes (CNs) produces sidelobes that are highly dependent on the collaborating nodes’ locations. The sidelobes cause interference and affect the communication rate of unintended receivers located within the transmission range. Nulling is not possible in an open-loop CBF since the collaborating nodes are unable to receive feedback from the receivers. Hence, the overall sidelobe reduction is required to avoid interference in the directions of the unintended receivers. However, the impact of sidelobe reduction on the capacity improvement at the unintended receiver has never been reported in previous works. In this paper, the effect of peak sidelobe (PSL) reduction in CBF on the capacity of an unintended receiver is analyzed. Three meta-heuristic optimization methods are applied to perform PSL minimization, namely genetic algorithm (GA), particle swarm algorithm (PSO) and a simplified version of the PSO called the weightless swarm algorithm (WSA). An average reduction of 20 dB in PSL alongside 162% capacity improvement is achieved in the worst case scenario with the WSA optimization. It is discovered that the PSL minimization in the CBF provides capacity improvement at an unintended receiver only if the CBF cluster is small and dense.

## 1 Introduction

Collaborative beamforming (CBF) is a promising scheme for the Internet of Things (IoT) and Machine to Machine (M2M) communications in the 5G standard [1]. In CBF, decentralized nodes act as a distributed transmit antenna array and adjust the initial phases of their carriers to form a beam collaboratively towards an intended receiver [2, 3]. It has been established that the CBF with *N* collaborating nodes transmitting at a fixed transmit power is capable of extending the transmission range by *N*. Similarly, the transmission power can be reduced by 1/*N*^2^ per node for a fixed transmission range [4].

The need for CBF arises from two distinct problems faced in the wireless sensor networks (WSN), namely the range and battery limitations. Various methods have been suggested in the past to solve these limitations. A strategic sensor deployment could result in efficient use of energy [5, 6]. However, this option is not viable for a dynamic network. Ahmadi et al. proposed an energy efficient routing algorithm in the WSN that maintains the coverage and reliability of the network [7]. However, the coverage is still omnidirectional and communication will still fail if there is a link failure between the source and the sink. Collaborative beamforming on the other hand, is directive and also enables isolated cluster to establish communication with efficient energy usage [8, 9].

Though the beampattern of the CBF is directive, sidelobes exist at directions other than the mainlobe. It has been noted in [4, 10] that the randomness of the nodes’ locations in the CBF results in high and asymmetrical sidelobes in the sample beampattern, especially when the number of collaborating nodes *N* is small. These sidelobes will interfere with communications of other unintended receivers and reduce the communication capacity of these receivers.

**Fig 1:**
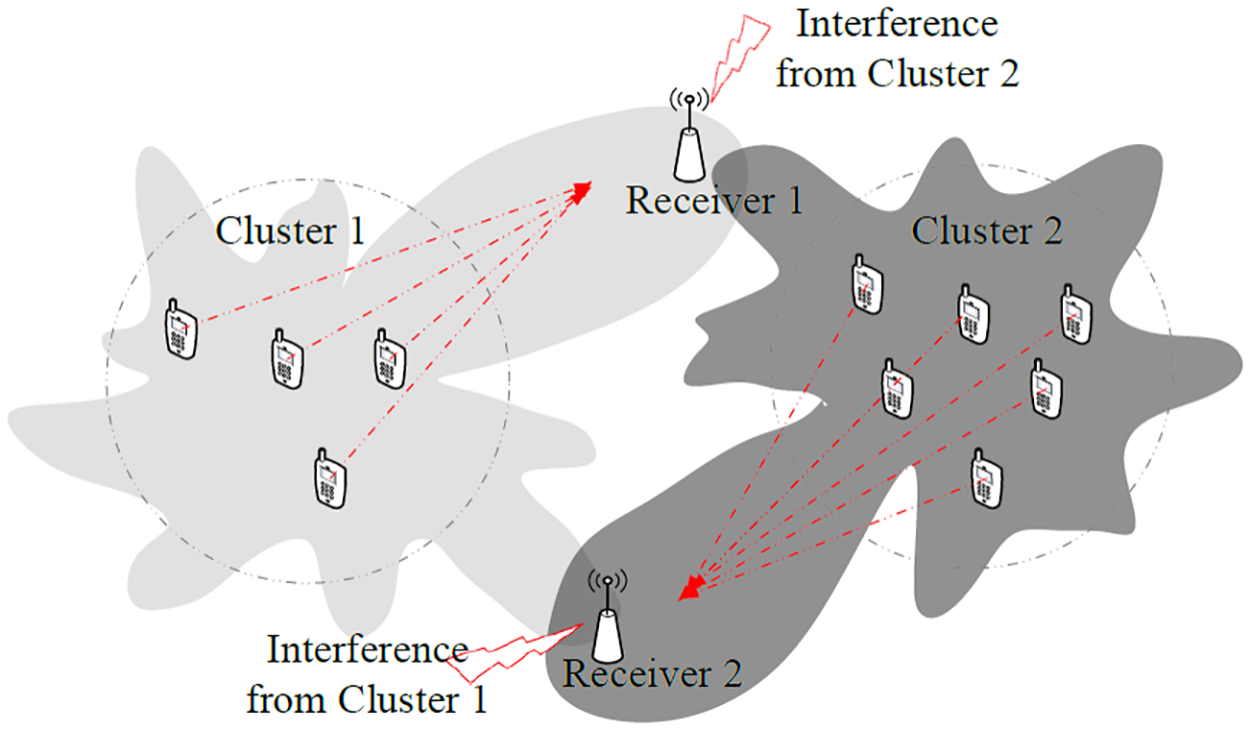
The effect of sidelobes on unintended receivers. The sidelobe of one CBF cluster interferes the communication of the other cluster.

To illustrate the damaging effect of sidelobes in the CBF transmissions, consider two clusters of WSNs each trying to establish a connection with remote receivers, as shown in Fig 1. While both clusters successfully beamform towards their respective receivers, the sidelobes of the beampatterns cause interference to each other. The interference reduce the receivers’ signal-to-interference-and-noise ratio (SINR) and thus results in degraded throughput. In a multi-channel network, optimal channel assignment can be performed to minimize the interference in the network [11]. However, if the network is sharing the same channel, the communication of the CBF has to be highly directive with limited sidelobes to avoid the interference phenomenon.

Effort to reduce the sidelobes in the CBF was first attempted in [12], where a sub-optimal greedy selection algorithm was proposed to select a subset of sensor nodes from the cluster. Unintended receivers experiencing interference due to the collaborative transmission of the cluster continuously send feedback to the cluster until the cluster selects the best subset that produces nulls at the directions of these receivers. Chen et. al proposed cross entropy optimization to solve the same problem [13]. However, these selection methods are only feasible when a large number of nodes is available in the cluster. On the other hand, [14] proposed an iterative phase perturbation scheme to simultaneously increase the power at the intended receiver and decrease the power (i.e. producing null) at the unintended receivers. However, the hitting time taken to achieve a good solution increases substantially as the number of unintended receivers increases.

The papers [12–14] all focus on null creation at the directions of the unintended receivers. The null creation, however, is only feasible in a closed-loop CBF, i.e., when the unintended receivers can send feedback to the collaborating nodes. This is a plausible assumption if the unintended receivers are the base stations (BSs) or access points (APs) on the same network. However, if the unintended receiver is a neighboring node with resource constraints, or is engaged in another communication, the feedback mechanism might not be possible. Furthermore, if the network is operating in an Industry, Scientific and Medical (ISM) band, the unintended receiver might be a node operating under a different standard and communication protocol and hence will not be able to send feedback. Therefore, in the open-loop CBF scenarios, where the unintended receivers are unable to provide feedback to the collaborating nodes, it is more useful to reduce the overall sidelobes rather than creating nulls at the specific directions of unintended receiver.

Nik et al. suggested the use of the particle swarm optimization (PSO) driven quasi-circular node selection method to reduce the sidelobe in the CBF [15]. Similarly, Sun et al. proposed a node selection method based on circular array using the firefly algorithm (FA) [16]. Both papers adopted node selection method to achieve sidelobe reduction, imitating the array synthesis method in the centralized array. However, in a cluster where the collaborating nodes’ positions are fixed and cannot be manipulated, node selection method may utilize some nodes in the cluster more often than others. Therefore, the node selection method tends to exhaust the energy of nodes frequently used for the CBF, which reduces the lifetime of the sensor nodes.

This paper proposes an amplitude selection method to reduce the sidelobes using metaheuristics optimization algorithms [17–19]. The conventional genetic algorithm (GA) and PSO, as well as a fairly new and simple swarm-based algorithm called the weightless swarm algorithm (WSA) [20, 21] are selected as the optimization tools. The new WSA algorithm is a simplified version of the legacy PSO with the capability to achieve equally powerful results.

Imperatively, this paper investigates the effect of sidelobe reduction on the capacity of a communication. The existing literature on sidelobe reduction in the CBF focuses only on the beampattern analysis and does not investigate the impact of the sidelobe reduction on the capacity of the unintended receiver. Ahmed et. al. briefly studied the significance of sidelobe control in improving the capacity of a network in [12]. However, the capacity analysis was build upon their proposed nulling scheme in a closed-loop CBF scenario and cannot be directly applied to analyze the capacity improvement gained with sidelobe reduction in an open-loop CBF. This paper bridges this research gap by providing an in-depth capacity analysis for the case of sidelobe reduction in an open-loop CBF. The results provide insights to how sidelobe reduction in the CBF affects the capacity of an unintended receiver for various cluster dimensions and the receive signal-to-noise ratio (SNR). Results further show that the WSA optimization algorithm provides better capacity at an unintended receiver compared to the legacy GA and PSO.

The rest of this paper is organized as follows. Section 2 describes the system model of the conventional CBF. In section 3, the optimization problem of the PSL reduction in the CBF as well as the capacity at unintended receivers are formulated. The GA, PSO and the WSA algorithms are detailed in Section 4. Results on the PSL and capacity improvement are presented in Section 4. Conclusions are provided in Section 5. Notation list defining all the symbols used in this paper is provided at the end of this paper.

## 2 System model

This section details the system model of a general collaborative beamforming in terms of array factor formulation and establishes the assumptions made in this paper.

### 2.1 Assumptions

A few assumptions are made throughout this paper. All these assumptions are commonly made in the literature [4, 12].

Since, resource limitation is prevalent at the sensor nodes and not the base station, only uplink beamforming is considered in this paper. For simplicity, it is assumed that all nodes and the receivers are of co-planar configuration, which is practical for a near-ground WSNs [12]. The idea can be easily extended to three dimensional CBF by considering the elevation angle when formulating the antenna array factor. For a network with a specific purpose such as monitoring or surveillance, all nodes are usually of the same model, hence it is assumed that all collaborating nodes are equipped with a single isotropic antenna with identical power constraint. The signals at all nodes are assumed to be synchronous which has been proven to be achievable in the previous works on the CBF [3, 22]. Static nodes are considered in this paper since they are more resource limited and will benefit from the CBF. Each node has the information of its own location coordinate as well as every other node in the cluster, which can be made known during the network configuration process.

### 2.2 Array factor

**Fig 2:**
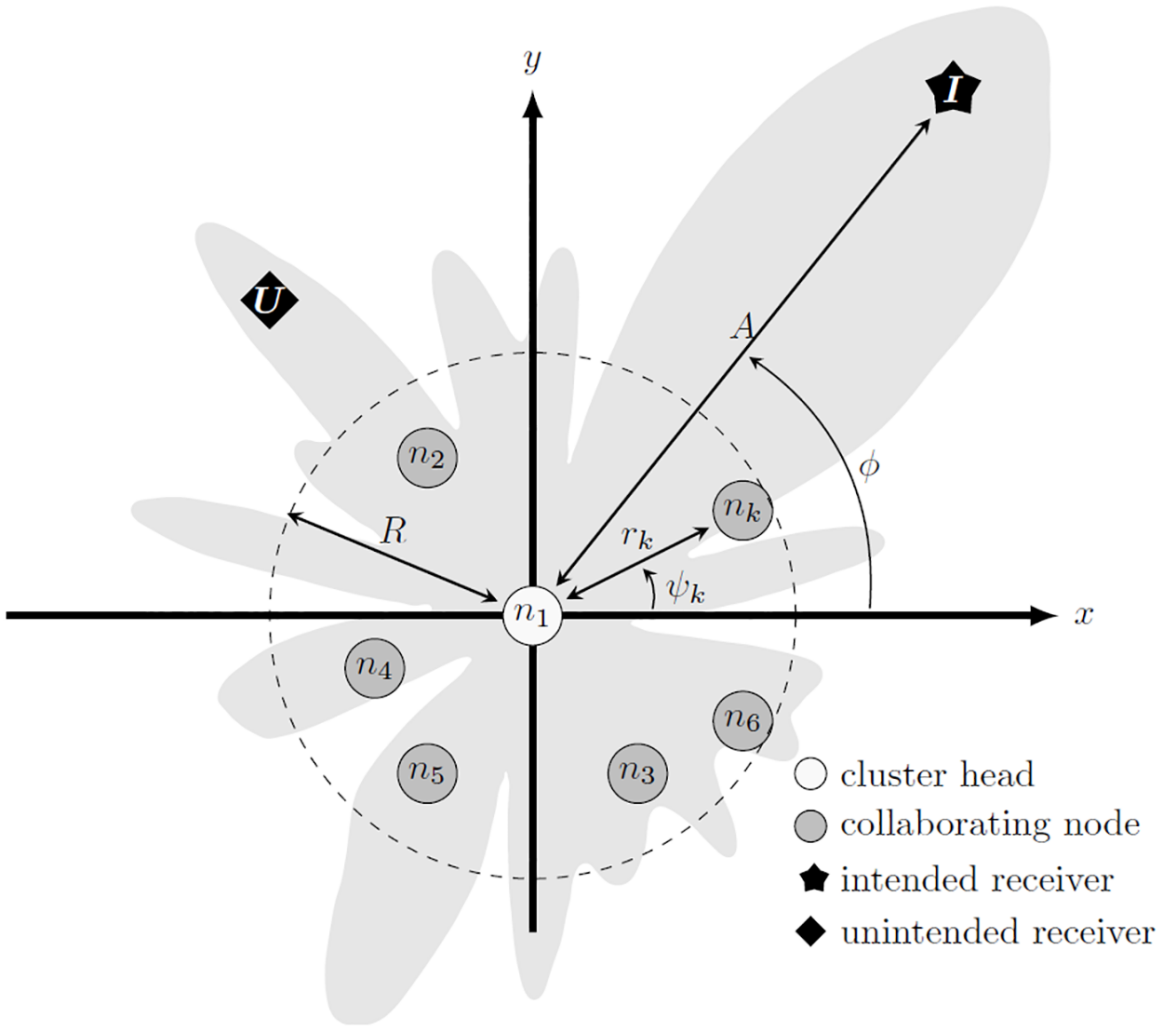
Geometrical model of collaborative beamforming [23].

A model with geometrical composition as detailed in Fig 2 is considered in this paper, where *N* nodes are scattered randomly within a cluster of the radius *R* meters on a two-dimensional plane.

The node locations are represented in polar coordinates where **r** = [*r*_1_, *r*_2_,…, *r*_*N*_] ∈ [0, *R*] and ***ψ*** = [*ψ*_1_, *ψ*_2_,⋯, *ψ*_*N*_] ∈ [− *π*, *π*]. The location of the node *k* = [1, 2, ⋯, *N*] is therefore denoted as (*r*_*k*_, *ψ*_*k*_). One of the nodes is chosen as the cluster head (CH), which becomes the geometrical reference point as well as the coordinator for all other nodes in the cluster. The CH processes the information of all nodes, performs the optimization and shares the optimized weight with all the collaborative nodes. The azimuth angle of the intended receiver with reference to the cluster head is *ϕ* ∈ [− *π*, *π*].

The array factor (AF) for the azimuth *θ* ∈ [− *π*,*π*] is approximated as [4]

$$\begin{array}{c} AF\left(\theta ,\xi \right)=\sum_{k=1}^{N}w_{k}e^{j\frac{2\pi }{\lambda }r_{k}\left[\cos\left(\theta -\psi_{k}\right)\right]} \end{array}$$ (1)

where *λ* is the wavelength of the signal and *w*_*k*_ is the *k*-th node’s transmission weight

$$\begin{array}{c} w_{k}=\xi_{k}e^{j\Psi_{k}} \end{array}$$ (2)

whereby *ξ* = {*ξ*_*k*_, *k* = 1, 2,…, *N*} is the transmission amplitude whereas **Ψ** = {*Ψ*_*k*_, *k* = 1, 2,…, *N*} is the initial phase of each collaborating node.

The conventional CBF technique follows the fundamental delay-and-sum beamformer concept. Weights of all the collaborating nodes have equal transmission amplitude, therefore *ξ*_*k*_ = 1 for *k* = [1, 2,⋯, *N*]. The beam is steered to a specified direction *ϕ* by selecting suitable phase *Ψ* for the weight of each node. In an open-loop scenario, the phase synchronization is done by compensating the distance between a collaborating node and the cluster head. As a result, the initial phase of node *k* in the conventional CBF is [4]

$$\begin{array}{c} \Psi_{k}=-\frac{2\pi }{\lambda }r_{k}\cos\left(\phi -\psi_{k}\right) \end{array}$$ (3)

Therefore, the conventional beamformer can accurately direct its main beam towards the intended destination when the positions of all the collaborating nodes are known. However, the sidelobe level of the beampattern cannot be controlled by applying the initial phase provided in Eq (3) as the weight.

## 3 Problem formulation

### 3.1 Communication model for the peak sidelobe optimization

Since the uplink transmission in the WSN is a bursty traffic, the system works well in a time-slotted transmission scheme [12]. The collaborating nodes and receiver will periodically exchange location information when the network is idle. Consequently, this paper proposes a four-stage communication procedure, given as follows:

1. The source node broadcasts data to all nodes in the cluster.
2. Nodes willing to participate in the communication respond using contention protocol. The cluster head processes the information to obtain the optimal weight combination for all collaborating nodes.
3. The cluster head shares the optimal weight with the collaborating nodes.
4. Each collaborating node adjusts its beamforming coefficient accordingly and simultaneously transmits the common data.

The cluster head has to perform the optimization process in a short period to avoid network latency.

### 3.2 Objective function

The optimization goal of this paper is to choose a combination of transmission amplitude **ξ** at the collaborating nodes such that the peak sidelobe of the CBF beampattern is minimized. Unlike phase and amplitude perturbation in [23], this paper attempts amplitude-only perturbation. The phase can be obtained using Eq (3) when the locations of the nodes are known and fixed.

The PSL optimization has to satisfy a few constraints. First, it is necessary to ensure that the mainbeam of the CBF is not shifted after the optimization. Hence, the first constraint *g*_1_ is an inequality that limits a pointing error to a predefined bound *β*. The pointing error is the difference between the look angle of the mainlobe and the true look angle.

$$\begin{array}{c} \begin{array}{c} g_{1}\left(\xi \right)=\left|\phi -\theta_{ML}\right| \end{array} \end{array}$$ (4)

In this work, *β* is set to $\frac{\pi }{180^{{}^{\circ}}}$, which accounts to a pointing error of 1°.

The look angle of the mainlobe is the argument of the maximum of the array factor:

$$\begin{array}{c} \begin{array}{c} \theta_{ML}=\text{arg}\;\text{max}\left|AF\left(\theta ,\xi \right)\right|,\;\theta \in \left[-\pi ,\pi \right] \end{array} \end{array}$$ (5)

The combined maximum transmit power, EIRP_max_ is limited to 36 dBm, according to the Federal Communications Commission (FCC) regulation for the ISM band. Additionally, the CBF transmission should satisfy a threshold received power *P*_rx(min)_ at the intended direction *ϕ* to ensure successful communication. In this paper, the *P*_rx(min)_ is set to 30 dBm.

Therefore, a second constraint *g*_2_(*ξ*) is introduced to ensure that the power of the beampattern produced by the CBF is within the maximum allowed effective isotropic radiated power (EIRP_max_) for the network and at the same time satisfies the threshold received power *P*_rx(min)_:

$$\begin{array}{c} \begin{array}{c} g_{2}\left(\xi \right)=\text{max}\left(20\;\;{\text{log}}_{10}\left\{AF\left(\theta ,\xi \right)\right\}\right);\theta \in \left[-\pi ,\pi \right] \end{array} \end{array}$$ (6)

The optimization problem thus can be defined as

$$\begin{array}{c} \begin{array}{cccc} & \underset{\xi }{\text{minimize}} & & f(\xi )\\ & \text{subject}\;\text{to} & & g_{1}\left(\xi \right)\leq \beta \\ & & & P_{\text{rx(min)}}\leq g_{2}\left(\xi \right)\leq EIRP_{\text{max}}\\ & & & \xi \in [0,\sqrt{P_{t}}] \end{array} \end{array}$$ (7)

where *P*_*t*_ is the maximum transmit power of the isotropic antenna at each collaborating node. The objective function *f*(*ξ*) is the normalized PSL in decibel

$$\begin{array}{c} \begin{array}{c} f\left(\xi \right)=20\;{\text{log}}_{10}\frac{\text{max}\;AF(\theta_{\text{SL}},\xi )}{AF(\theta_{ML},\xi )} \end{array} \end{array}$$ (8)

The numerator in the Eq (8) represents the highest peak value of the sidelobes *θ*_**SL**_. The positions of *θ*_**SL**_ is identified by finding all the peak points of the array factor (other than the mainlobe’s peak) for the domain *θ* ∈ [− *π*, *θ*_*ML*_) ∪ (*θ*_*ML*_, *π*] such that the argument *θ* satisfies $(\frac{\partial }{\partial \theta }AF\left(\theta ,\xi \right)=0)$ and $\left(\frac{\partial^{2}}{\partial \theta^{2}}AF\left(\theta ,\xi \right)<0\right)$.

The optimization function is a non-convex problem since $\frac{\partial }{\partial \theta }AF\left(\theta ,\xi \right)=0$ is intractable. Heuristic methods are preferred to solve the problem as the best solution can be obtained much faster. In this paper, population-based meta-heuristic methods are considered as the solutions since such algorithms are more effective and converge faster due to the parallel processing of candidate solutions.

### 3.3 SINR and capacity at the unintended receiver

To analyze how the optimized beampattern affects the capacity of an unintended receiver, the channel model and the receive SINR have to be formulated. Each collaborating node in the CBF transmits common data symbol **s** using an isotropic reciprocal antenna with the transmit power *P*_*k*_ = (*ξ*_*k*_)^2^. Hence, from the array factor Eqs (1)–(3), the corresponding received signal **y** at a far field receiver RX, located at an arbitrary direction *θ* ∈ [− *π*,*π*] is

$$\begin{array}{c} \begin{array}{c} y\left(\theta \right)=\sum_{k=1}^{N}\xi_{k}h_{X_{{\text{RX}}_{k}}}e^{j\frac{2\pi }{\lambda }r_{k}\left[\cos\left(\theta -\psi_{k}\right)-\cos\left(\phi -\psi_{k}\right)\right]}s+w \end{array} \end{array}$$ (9)

where $w∼CN(0,\sigma_{w_{{\text{RX}}_{k}}}^{2})$ is the additive white Gaussian noise (AWGN) at receiver RX and *h*_RX_*k*__ is the channel coefficient between the *k*^*th*^ node and the RX. The channel coefficient is a product of the fading gain *a*_RX_*k*__ and the attenuation effect *b*_RX_*k*__ due to the propagation distance between node *k* and the RX. Thus *h*_RX_*k*__ = *a*_RX_*k*__ *b*_RX_*k*__.

The angle at the direction of the intended receiver is *θ* = *ϕ*. Hence, from Eq (9), the SNR at the intended receiver I is

$$\begin{array}{c} \begin{array}{c} {\text{SNR}}_{I}\left(\xi \right)=\frac{\sum_{k=1}^{N}{||\xi_{k}h_{I_{k}}||}^{2}}{\sigma_{w_{I}}^{2}} \end{array} \end{array}$$ (10)

To gauge the capacity improvement at an unintended receiver U, the channel effect should also be considered. Since a near ground WSN application is considered, a time-invariant channel with predominantly large-scale fading is assumed [12]. Specifically, a log-normal distributed random variable is assumed for the channel, where $a_{I_{k}}∼\exp\left[N\left(0,\sigma_{a}^{2}\right)\right]$, where $\sigma_{a}^{2}=0.2$. Since the distance between the collaborating nodes and the receiver is far greater than the distance between the collaborating nodes, it can be assumed the path loss component is identical for all the nodes such that *b*_I_*k*__ = 1 for *k* = {1, 2,…*N*}.

The corresponding capacity at an unintended receiver located at *ϕ*_*U*_ can be calculated if the input SNR at the unintended receiver is known. Provided that the receiver *U* receives a useful signal at the power *γ*^2^, the SNR is $\gamma^{2}/\sigma_{w_{U}}^{2}$, where $\sigma_{w_{U}}^{2}$ is the variance of the AWGN at *U*. If the communication is interfered by a far field CBF, the SINR at the unintended receiver *U* becomes

$$\begin{array}{c} \begin{array}{c} SINR_{\text{U}}=\frac{\gamma^{2}}{\varepsilon +\sigma_{w_{U}}^{2}} \end{array} \end{array}$$ (11)

where

$$\begin{array}{c} \begin{array}{c} \varepsilon =\sum_{k=1}^{N}{||\xi_{k}h_{U_{k}}e^{j\frac{2\pi }{\lambda }r_{k}\left[\cos\left(\phi_{U}-\psi_{k}\right)-\cos\left(\phi -\psi_{k}\right)\right]}||}^{2} \end{array} \end{array}$$ (12)

With the SINR information, the capacity *C* at the unintended receiver is

$$\begin{array}{c} \begin{array}{c} C={\text{log}}_{2}\left\{1+SINR_{\text{U}}\right\} \end{array} \end{array}$$ (13)

**Fig 3:**
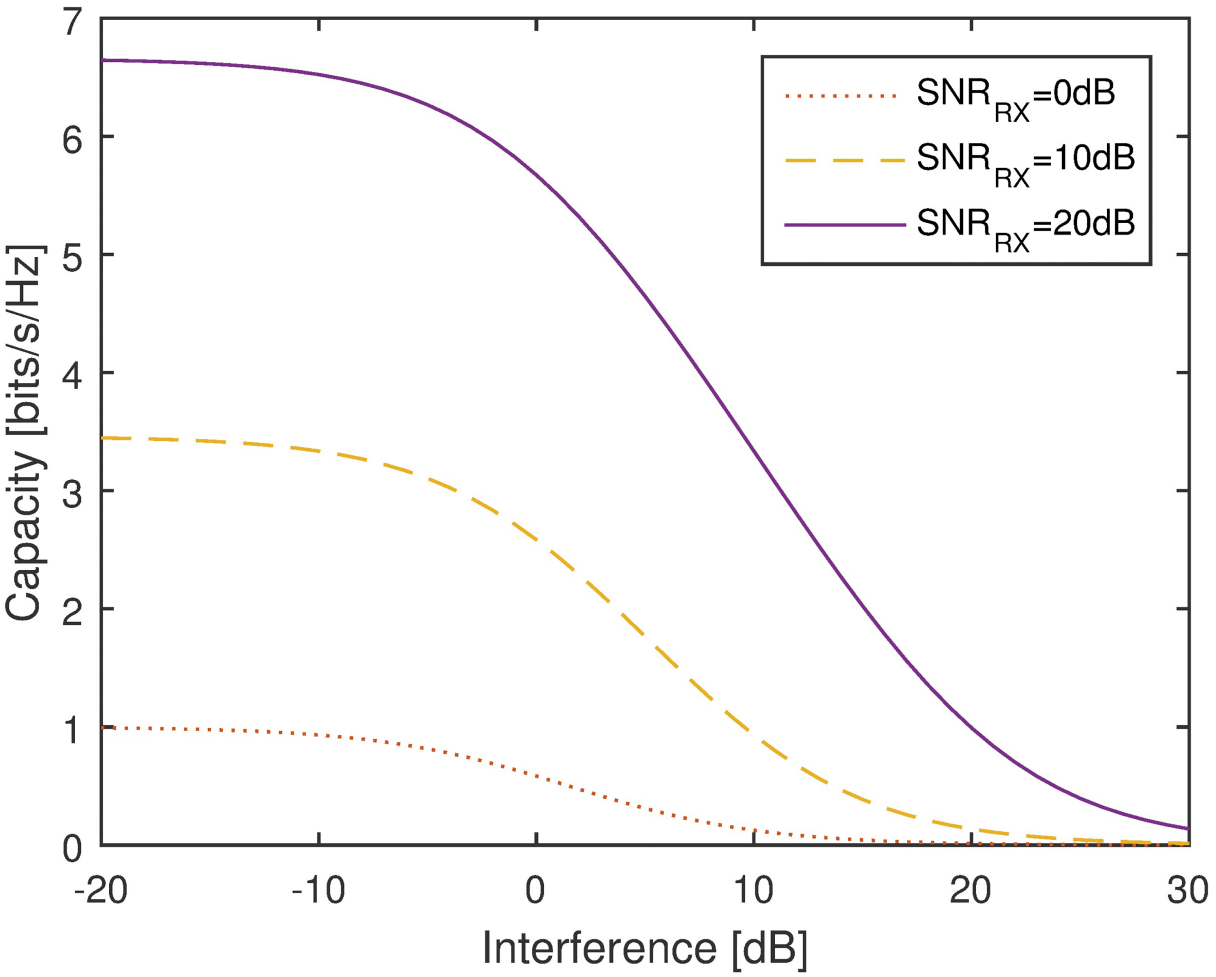
Capacity vs. interference level at a receiver with receive SNR = {0, 10, 20}.

Fig 3 shows a quantitative representation of how an interference could affect the capacity of a receiver operating at different values of the receive SNR. Note that an interference level as low as 0dB causes a significant performance drop. Therefore, it is indeed necessary to reduce the sidelobes of the CBF to ensure that interference to unintended receivers are limited.

## 4 Summary of optimization algorithms

In this paper, population-based meta-heuristics are chosen as candidate solutions to solve the optimization problem. The genetic algorithm (GA) and the particle swarm optimization (PSO) are considered in this paper due to the reliability of these algorithms. Although the results obtained via meta-heuristics are sub-optimal and not the optimal solutions, both GA and PSO have the reputation of being able to provide solutions close to the optimal solutions in the long run. A relatively new algorithm called the weightless swarm algorithm (WSA) is also considered in this paper due to the simplicity of the algorithm compared to the legacy methods.

### 4.1 Genetic algorithm

The genetic algorithm (GA) is an optimization method based on the principles of genetics and the survival of the fittest theorem. According to this theorem, only organisms with the good genetic traits will be promoted to the next generation via natural selection. Each input string is treated as a chromosome of an individual.

For the GA optimization tool in this paper, the initial population of genes is generated for *M* number of chromosomes. Each chromosome will contain an *N* number of genes. The fitness function for each chromosome is evaluated and *ρM* of the best-ranked chromosomes are chosen as parents for the next generation, where *ρ* ∈ [0, 1] is the parent selection ratio. A crossover point is randomly chosen in each gene of the parents, and the part of chromosomes beyond and after the crossover points is swapped with the chromosomes of another parent to generate *M* − 1 offsprings. Next, mutation process randomly changes the values of a few chromosomes in the offspring to a random value within the feasible region, based on pre-set mutation rate value *μ* ∈ [0, 1]. In an attempt to preserve the elite, the parent with the best rank is retained as the offspring of the next generation. The *M* offspring population is evaluated based on the fitness function and ranked in the next generation. The algorithm is stopped when it reaches the maximum number of generation *I*. The pseudocode of the GA is presented in Algorithm 1.

**Algorithm 1** Genetic Algorithm

1: Generate M chromosomes each with N genomes

2: **while** *i* < *I* **do**

3:  **for** each chromosome **do**

4:   calculate fitness function ← *f*

5:  sort *f*

6:  choose *ρM* solutions as parents

7:  swap the values of parents to generate *M* − 1 new chromosomes as new candidates

8:  mutate *μNM* values in the population to a random value

9:  append chromosome with the best *f* as the *M*-th chromosome

### 4.2 Particle swarm optimization

The particle swarm optimization (PSO) is a popular metaheuristic optimization method due to its straight forward approach and low computation load memory. Each input string is considered as a particle in this method. A particle at iteration *i* will have a position *x*^*i*^ and velocity *v*^*i*^. The best position achieved by the particles in iteration *i* is defined as the local best $x_{p_{best}}^{i}$ and the best $x_{p_{best}}^{i}$ among all iterations is considered as the global best position *x*_*g*_*best*__. Every particle will achieve a better position by updating its current velocity and position according to its previous position and the previous best positions of the particles [24]. The formulation of updating the velocity of an *n*-th particle at *i*-th iteration is [25]

$$\begin{array}{c} \begin{array}{cc} v_{n}^{i+1}= & \left[\omega \times v_{n}^{i}\right]+\left[c_{1}\times rand\left(\right)\times \left(x_{g_{best}}-x_{n}^{i}\right)\right]\\ & +[c_{2}\times rand\left(\right)\times \left(x_{p_{best}}^{i}-x_{n}^{i}\right)] \end{array} \end{array}$$ (14)

the position of the *n*-th particle for the next iteration is updated such that

$$\begin{array}{c} x_{n}^{i+1}=x_{n}^{i}+v_{n}^{i+1} \end{array}$$ (15)

where *c*_1_ is the cognitive parameter *c*_2_ is the social parameter *ω* is the inertia weight index and *rand*() is a random value within the [0, 1] range. The ratio of *c*_1_: *c*_2_ will determine the importance given to local best and global best. The inertia weight *ω*, regulates impact of the previous velocity on the new velocity during updates. The algorithm is stopped after reaching the maximum iteration *I*. The pseudo-code of the PSO is presented in Algorithm 2.

**Algorithm 2** Particle Swarm Optimization

1: Generate *M* particles each with *N* values ← *x*^*M*x*N*^

2: **while** *i* < *I* **do**

3:  **for** each particle **do**

4:   calculate fitness function ← *f*

5:  find the best value if *f* ← *f*_*best*_(*i*)

6:  identify particle with local best solution ← *p*_*best*_(*i*)

7:  **if** *f*_*best*_(*i*) < *f*_*best*_(*i* − 1) **then**

8:   update global best solution ← *p*_*best*_(*i*)

9:  **for** each particle **do**

10:   update velocity ← *v*

11:   update particle ← *x*

### 4.3 Weightless swarm algorithm

The weightless Swarm Algorithm (WSA) is a recent swarm-based algorithm, introduced by Ting et al. in 2012 [20, 21]. In the WSA, several parameters prominent in the PSO are omitted. The well-known inertia weight *ω* is not present. Hence, it means that the velocity *v* is also unnecessary. Without *v*, a user also discards the concern of the bounds for this parameter, namely *v*_max_ and *v*_min_. Thus, the proposed algorithm has a much simpler form compared to the canonical PSO, where

$$\begin{array}{c} x_{n}^{i+1}=x_{n}^{i}+c_{1}\times rand\left(\right)\times \left(x_{g_{best}}-x_{n}^{i}\right) \end{array}$$ (16)

The local best solution is retained and carried forward to the next iteration to preserve the best solution. Compared to the legacy PSO algorithm, the complexity is significantly reduced, and only one parameter, i.e. *c*_1_ requires tuning. The pseudo-code of WSA is presented in Algorithm 3.

**Algorithm 3** Weightless Swarm Algorithm

1: Generate *M* particles each with *N* values ← *x*^*M*x*N*^

2: **while** *i* < *I* **do**

3:  **for** each particle **do**

4:   calculate fitness function ← *f*

5:  find the best value if *f* ← *f*_*best*_(*i*)

6:  identify particle with local best solution ← *p*_*best*_(*i*)

7:  **if** *f*_*best*_(*i*) < *f*_*best*_(*i* − 1) **then**

8:   update global best solution ← *p*_*best*_(*i*)

9:  **for** each particle **do**

10:   **if** particle ≠ *p*_*best*_ **then**

11:    update particle ← *x*

Table 1 compares the tuning parameters and the space-time complexities of the GA, PSO and WSA. It can be seen that the PSO and WSA are less computationally extensive compared to the legacy GA. Note that while the PSO and WSA have the same space-time complexity, the tuning of the WSA is much easier as only *c*1 has to be tuned compared to three parameters for the PSO.

**Table 1:** Comparison of the GA, PSO and WSA.

	GA	PSO	WSA
tuning parameters	*μ*, *ρ*	*c*1, *c*2, *ω*	*c*1
time complexity	$O(MN+M^{2})$	O(MN)	O(MN)
space complexity	$O(MN+M^{2}+M)$	O(N(M+1))	O(N(M+1))

## 5 Simulation results and discussions

In this section, the properties of the optimized beampattern and consequently the effects of the optimized beampattern on the capacity of an unintended receiver are analyzed. The PSL and the half-power bandwidth (HPBW) of the optimized beampattern are recorded for various sizes and node densities of a CBF cluster. The comparisons of the proposed optimization algorithms in terms of convergence rate are also presented to identify the algorithm with the fastest response. Capacity analysis is presented for three cases: 1) capacity for the sample CBF beampatterns, 2) capacity in worst case scenario, and 3) average capacity. The results provide insights on how sidelobe reduction affects the capacity of an unintended receiver within the transmission range of the CB for various dimensions of clusters and the receiver’s signal-to-noise ratio (SNR).

Simulation results presented in this section are performed with the aid of the MATLAB software. All angular directions are mentioned in degree (°) for better readability, though the values are in radians when implemented in the array factor equations. The radius of the collaborating nodes’ cluster *R*, is normalized so that $\tilde{R}=R/\lambda$. Without the loss of generality, the look angle of the intended receiver I is fixed at *ϕ* = 0°. The location of the unintended receiver U is an arbitrary value chosen from a uniform probability density function (pdf) between $\phi_{U}∼U\left[-180^{{}^{\circ}},180^{{}^{\circ}}\right]$. To limit the latency caused by the optimization process, the maximum iteration *I* for GA, PSO and WSA are all limited to *I* = 100 with population size *M* = 20. For each case analysed in this paper, average results are obtained from Monte Carlo simulations of 250 randomly placed *N* nodes within the radius $\tilde{R}$.

### 5.1 Parameter selection in optimization algorithms

It has been proven in the no free lunch (NFL) theorem that an ultimate meta-heuristic method that works for all optimization problem does not exist [26]. The parameters in an optimization algorithm have to be chosen carefully to ensure that the optimization tool could provide the best possible solution for a problem. There are two parameters in GA, namely selection rate *ρ* and mutation rate *μ*. Considering each parameter is analyzed with step size 0.1, the size of the search space is 100. Similarly, for the PSO, for restrictions of *c*_1_ ∈ [0, 4], *c*_2_ ∈ [0, 4] and *ω* ∈ [0, 1.5] with step size of 0.1 for all three parameters, the size of the search space is 24000.

To select the best parameter values, a parameter sensitivity analysis (PSA) that maps the output of every combination of the parameters is necessary. Each parameter has to be tested in a specific range with at least 30 repetitions to obtain the combination of parameter values that provides the best average output [23]. In this paper, each sample is repeated 50 times for each of the GA, PSO and WSA parameter combinations.

The parameter sensitivity analysis of the GA for sample *N* = 16, $\tilde{R}=1$, averaged from 50 runs, is presented in Table 2. There are two parameters in GA, namely selection rate *ρ* and mutation rate *μ*. Each entry in the table represents the peak sidelobe value for the specific combination of *ρ* and *μ*. For the restriction of *ρ* ∈ [0, 1], *μ* ∈ [0, 0.9] with step sizes 0.1 and 0.2, respectively, the best parameter combination GA is identified as *ρ* = 0.5, *μ* = 0.1.

**Table 2:** Parameter sensitivity analysis of the GA for sample *N* = 16, $\tilde{R}=1$.

*ρ*	*μ* = 0.1	*μ* = 0.3	*μ* = 0.5	*μ* = 0.7	*μ* = 0.9
0.1	-11.94	-13.51	-13.18	-14.68	-14.16
0.2	-11.56	-14.81	-11.51	-13.04	-15.47
0.3	-14.99	-13.46	-12.12	-12.16	-12.17
0.4	-14.54	-13.43	-13.18	-12.23	-11.69
0.5	**-16.76**	-15.85	-13.63	-12.64	-12.80
0.6	-13.72	-14.27	-14.09	-13.36	-13.68
0.7	-14.81	-15.06	-12.64	-12.98	-12.26
0.8	-13.79	-12.46	-13.28	-13.62	-16.69
0.9	-13.36	-14.91	-13.27	-14.28	-15.99
1	-13.13	-12.29	-12.53	-12.33	-12.44

Similarly, the parameter sensitivity analysis of the PSO is presented in Table 3. Each entry in the table represents the peak sidelobe value for the specific combination of *c*_1_, *c*_2_ and *ω*. The restrictions of *c*_1_ ∈ [0, 4] with a step size 0.1, and *c*_2_ ∈ [0, 4], *ω* ∈ [0, 1.5], with step sizes 0.5 for both, where *c*_1_ + *c*_2_ = 4, as suggested in [27], are adapted. The lowest PSL is achieved when *c*_1_ = 2, *c*_2_ = 2, and *ω* = 0.5, and hence these parameters are chosen for subsequent simulations.

**Table 3:** Parameter sensitivity analysis of the PSO for sample *N* = 16, $\tilde{R}=1$.

*ω*	*c*_1_ = 0.5	*c*_1_ = 1	*c*_1_ = 1.5	*c*_1_ = 2	*c*_1_ = 2.5	*c*_1_ = 3	*c*_1_ = 3.5
	*c*_2_ = 3.5	*c*_2_ = 3	*c*_2_ = 2.5	*c*_2_ = 2	*c*_2_ = 1.5	*c*_2_ = 1	*c*_2_ = 0.5
0.1	-12.82	-11.94	-12.90	-12.73	-11.66	-12.50	-13.05
0.2	-13.15	-12.29	-12.17	-12.23	-11.71	-12.27	-13.73
0.3	-13.93	-12.79	-13.89	-12.97	-13.17	-12.53	-13.76
0.4	-12.59	-13.60	-11.71	-13.44	-12.25	-14.40	-12.15
0.5	-11.83	-13.27	-13.27	**-14.91**	-12.28	-11.76	-11.79
0.6	-13.14	-11.81	-12.17	-12.20	-11.65	-11.04	-11.68
0.7	-11.50	-11.18	-10.53	-11.64	-11.70	-11.55	-11.15
0.8	-10.95	-11.72	-11.65	-11.28	-12.36	-11.14	-10.49
0.9	-10.96	-11.07	-11.37	-11.13	-10.32	-11.24	-10.34
1	-10.91	-10.42	-10.88	-11.23	-10.70	-10.65	-11.07

Only one parameter needs adjustment in the WSA compared to three parameters in the GA and PSO algorithm. Since the WSA exclude *ω* and *c*_2_, only the cognitive parameter *c*_1_ has to be tuned. Hence, the search space is reduced when compared to the PSO and this greatly simplifies the parameter sensitivity analysis.

**Fig 4:**
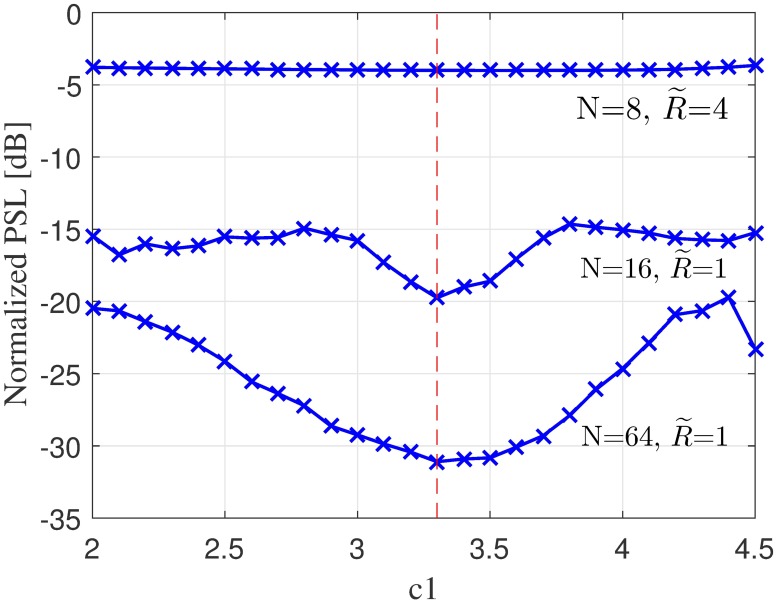
Parameter sensitivity analysis of the proposed WSA.

The WSA algorithm is applied to solve the problem for *c*_1_ ∈ [2, 4.5], with a step size 0.1. The average normalized PSL values are mapped to *c*_1_, as shown in Fig 4 where the minimum value is obtained for *c*_1_ = 3.3. To ensure that the best *c*_1_ value is consistent regardless of the dimension of the collaborating nodes’ cluster, the algorithm is tested for three different samples, which were *N* = 8, $\tilde{R}=4$, *N* = 16, $\tilde{R}=1$ and *N* = 64, $\tilde{R}=1$. The average normalized PSL values are minimum at *c*_1_ = 3.3 for the three examples and hence this value is adopted as the value of the parameter *c*_1_ in the WSA optimization algorithm.

### 5.2 Beampattern analysis

**Fig 5:**
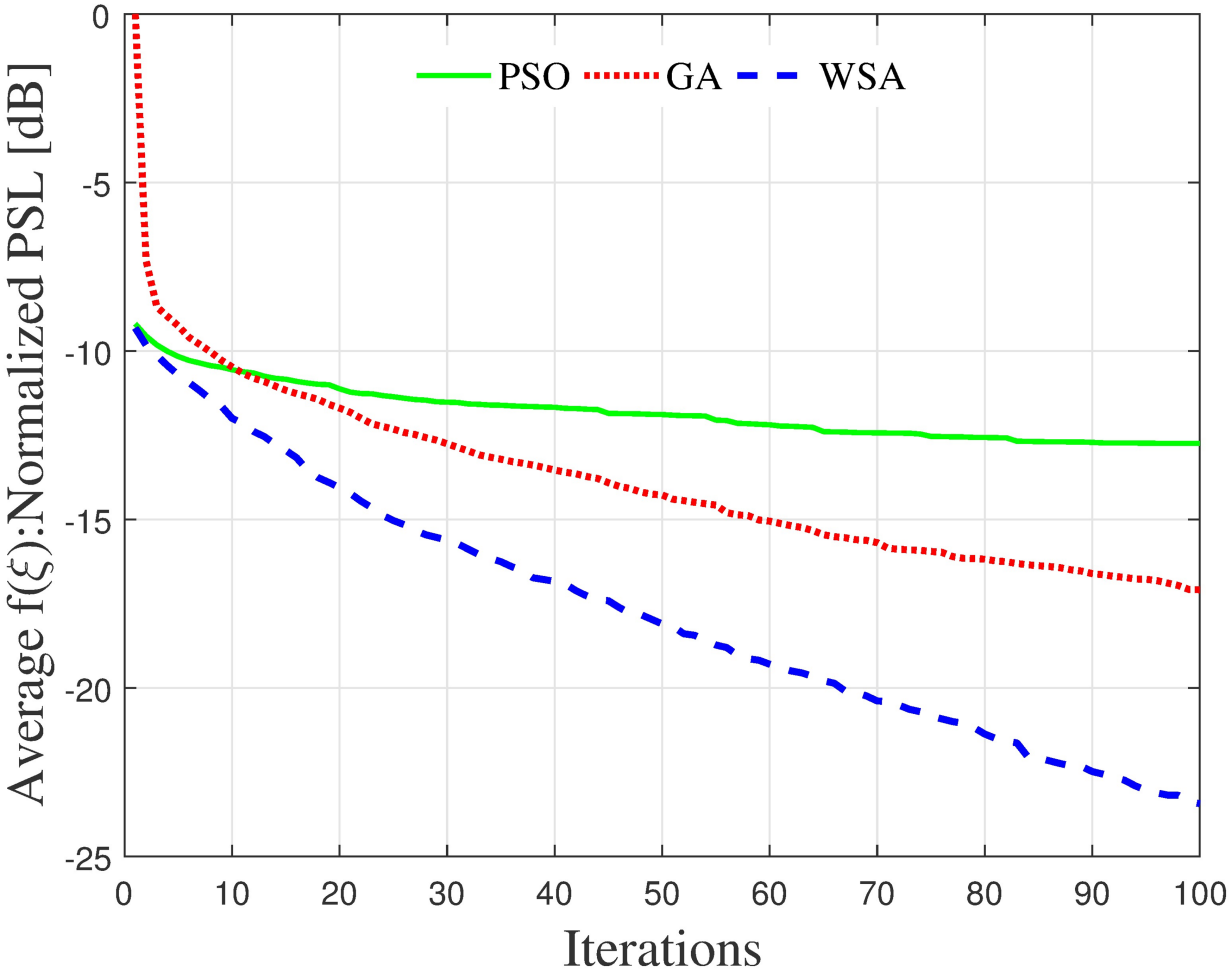
Mean convergence of fitness a cluster of *N* = 16 nodes and $\tilde{R}=1$ radius.

Fig 5 shows the average convergence of the fitness function for the CBF performed on *N* = 16 nodes, distributed uniformly within a disk size $\tilde{R}=1$ using the GA, PSO and WSA optimization. While the fitness of the PSO converges to a normalized PSL of −13 dB by the 100^th^ iteration, the results obtained with the GA and WSA achieves −17 dB and −23.5 dB respectively, promising further improvement if the iterations are increased. On average, the PSL obtained using the WSA algorithm is 6.5 dB lower with a faster convergence rate compared to the results obtained using GA algorithm.

**Fig 6:**
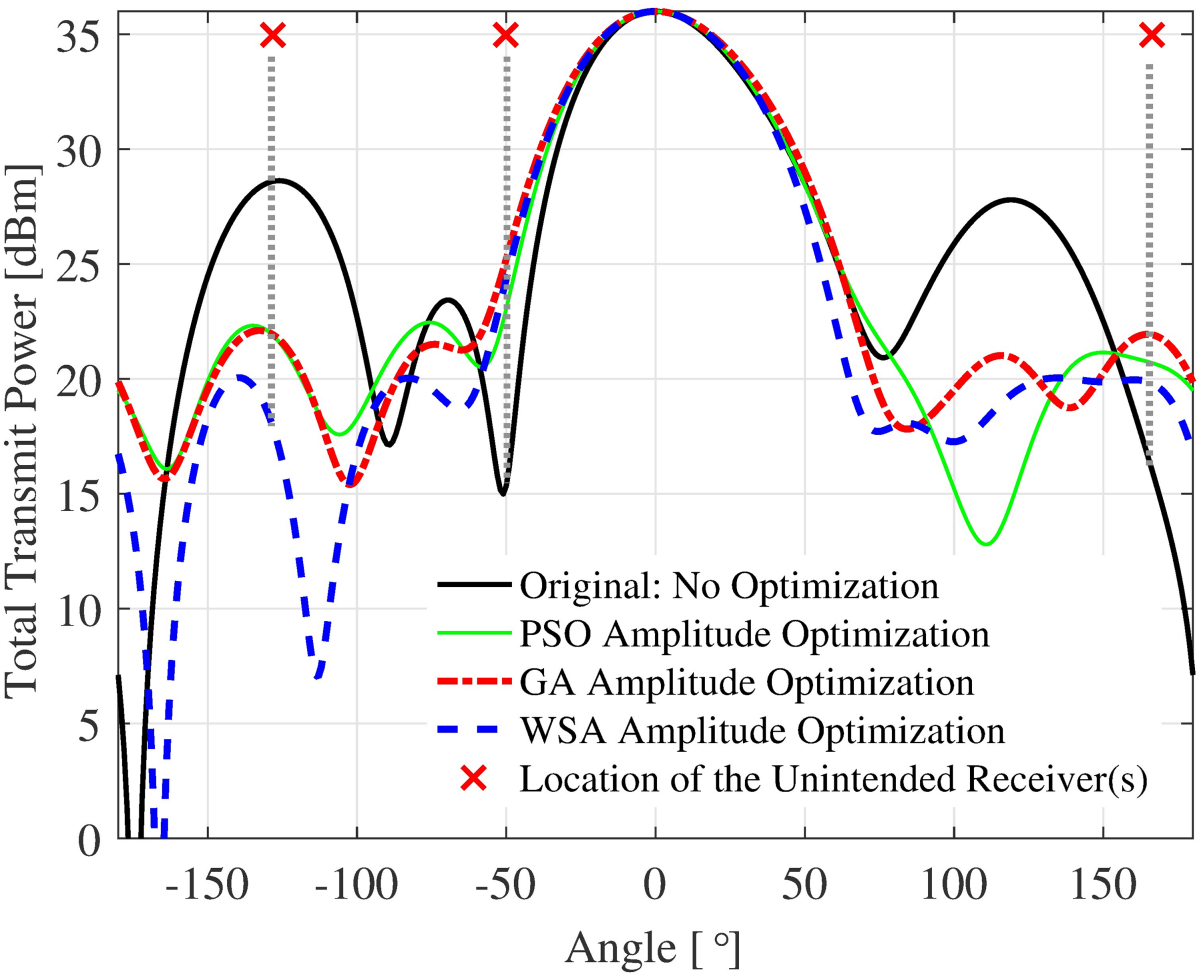
Sample beampattern for a cluster of *N* = 16 nodes and $\tilde{R}=1$ radius.

**Fig 7:**
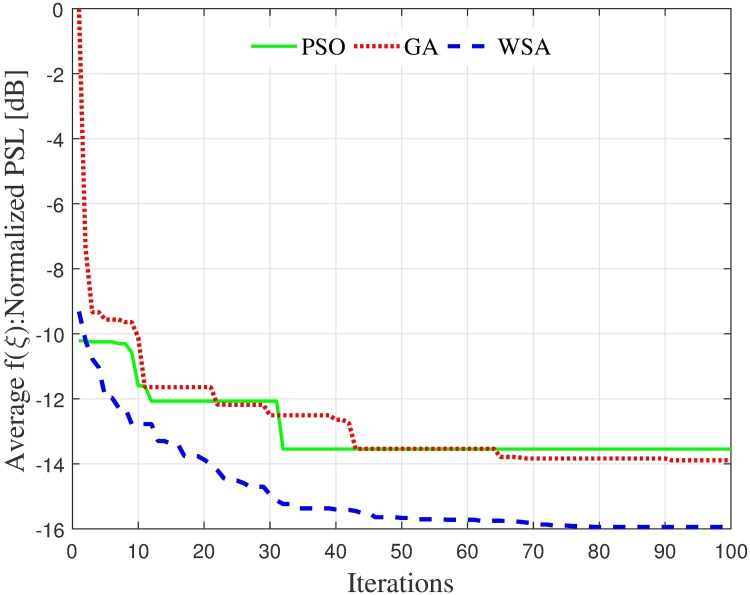
Sample convergence of fitness for a cluster of *N* = 16 nodes and $\tilde{R}=1$ radius.

Since the fitness function in the formulation is the PSL, and the PSL value is random and dependent on the node distribution [4], the average fitness convergence might not adequately gauge the efficiency of the optimization methods, as it does not reflect the individual PSL of a beampattern. Therefore, a sample is chosen from the 250 simulation samples to verify the beampattern and the fitness convergence, shown in Figs 6 and 7, respectively. For this particular sample, the final fitness value of the WSA algorithm is 6.5 dB better than the GA algorithm. The nodes’ positions for the sample shown in Fig 6 and the corresponding acquired amplitude *ξ* via each optimization method are summarized in Table 4 for the purpose of reproduction.

**Table 4:** Parameter values for sample Fig 6; the position and the optimized transmit amplitude of each node.

	position		*ξ*_*k*_			
*n*_*k*_	$\tilde{r_{k}}\left[\lambda \right]$	*ψ*_*k*_[rad]	original	GA	PSO	WSA
**2**	**0.228**	**1.742**	0.125	0.107	0.142	0.219
**3**	**0.150**	**-1.692**	0.125	0.000	0.042	0.003
**4**	**0.280**	**-2.106**	0.125	0.044	0.051	0.001
**5**	**0.064**	**1.007**	0.125	0.073	0.029	0.177
**6**	**0.222**	**0.180**	0.125	0.193	0.166	0.027
**7**	**0.178**	**-0.456**	0.125	0.031	0.042	0.032
**8**	**0.163**	**-1.606**	0.125	0.030	0.033	0.102
**9**	**0.101**	**-2.495**	0.125	0.083	0.049	0.221
**10**	**0.314**	**2.260**	0.125	0.295	0.238	0.308
**11**	**0.025**	**-2.367**	0.125	0.267	0.310	0.306
**12**	**0.112**	**2.538**	0.125	0.288	0.306	0.264
**13**	**0.172**	**-2.208**	0.125	0.050	0.048	0.055
**14**	**0.332**	**-2.758**	0.125	0.217	0.087	0.144
**15**	**0.054**	**1.228**	0.125	0.111	0.194	0.033
**16**	**0.057**	**0.518**	0.125	0.061	0.117	0.069

A few interesting observation can be made from the sample beampattern shown in Fig 6. First, while all the optimization methods provide lower PSL compared to the non-optimized beampattern, the location of the PSL are not fixed at the same location as with the non-optimized beampattern. In this sample, the PSL is initially located at −128° with 28.5 dBm transmit power. Both PSO and GA methods successfully reduce the PSL to about 28.5 dBm, however the position of the PSL is now at −135°. Similarly, the WSA optimization method shifts the PSL to −130° with a reduced value of 20 dBm. The shift in the PSL location is caused by the randomness of the nodes’ locations and the unique combination of **ξ** for different optimization methods used, which causes output beampattern to differ.

The second observation is that the beamwidth of the mainbeam is slightly widened when any of the optimization algorithms is employed. This phenomenon is common for all sidelobe reduction method using amplitude tapering [28]. Since the expansion of the beamwidth is only minimal, its damaging effect is minimal in general. However, if an unintended receiver is located very close to the mainbeam, for example, at −50° as shown in Fig 6, the unintended receiver will receive the interference from this beampattern with a gain of about 10 dB when all three optimization methods are used.

Finally, it can be observed that while the overall power at look angle *θ* ≠ *ϕ* of the optimized beampattern is much lower than the non-optimized beampattern, the optimized beam’s transmit power has the tendency to exceed the non-optimized transmit power at a few random locations. For example, it can be seen that at 166°, the GA, PSO and WSA all exceed the transmit power of the non-optimized beampattern by 5.5 dB, 4 dB and 3 dB, respectively. It can be concurred that reducing the PSL of a beampattern does not guarantee lower power at all other locations. However, given the limitation that the unintended nodes are not able to send useful feedback to the collaborating nodes, the PSL optimization is the best solution to reduce the transmit power at locations other than the intended receivers for the open-loop CBF. The sample beampattern confirms that barring a few locations such as −50° and 167°, the overall transmit power obtained via optimization is usually lower at *θ* ≠ *ϕ*.

**Fig 8:**
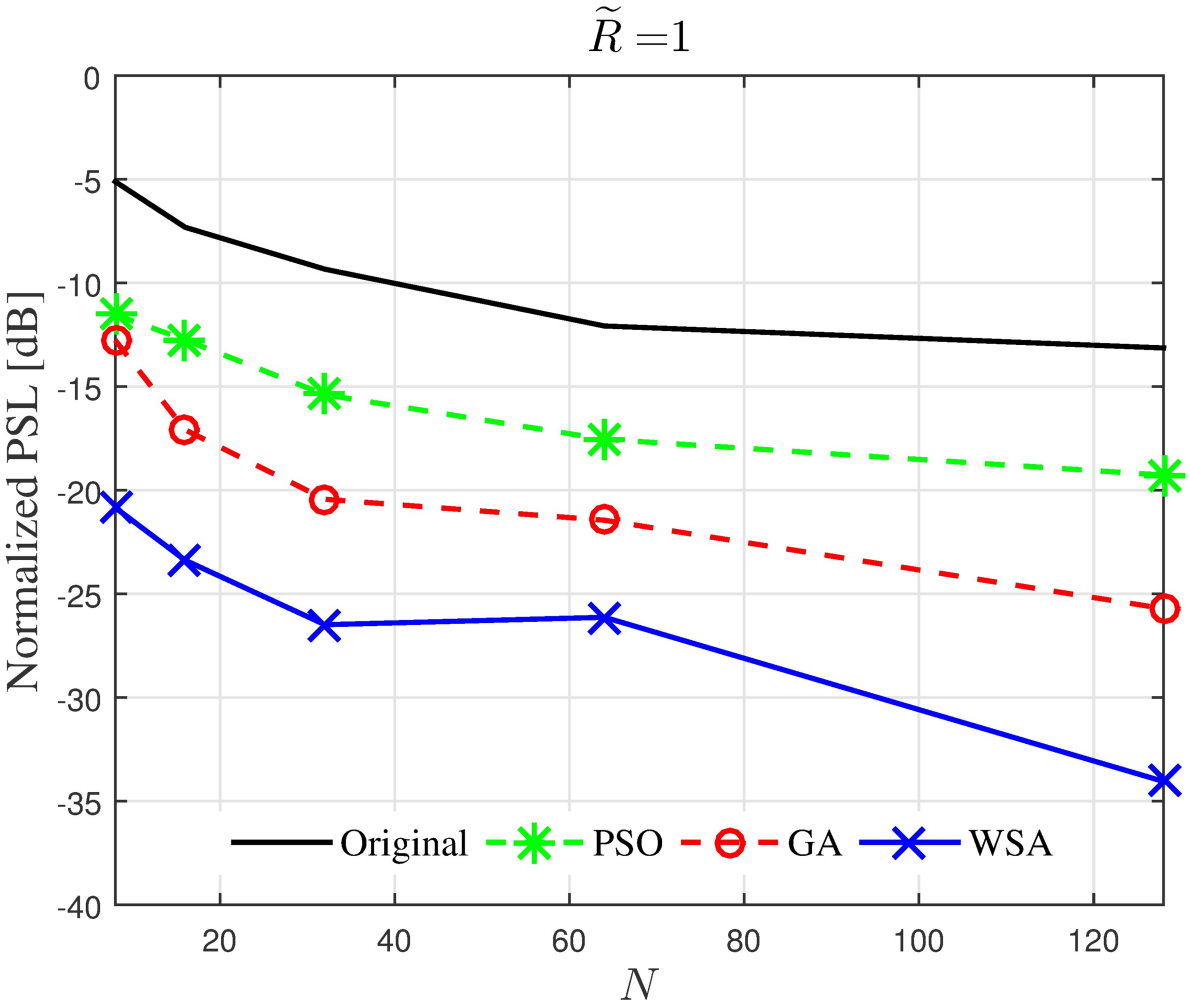
Comparisons of the PSL when *N* is varied.

**Fig 9:**
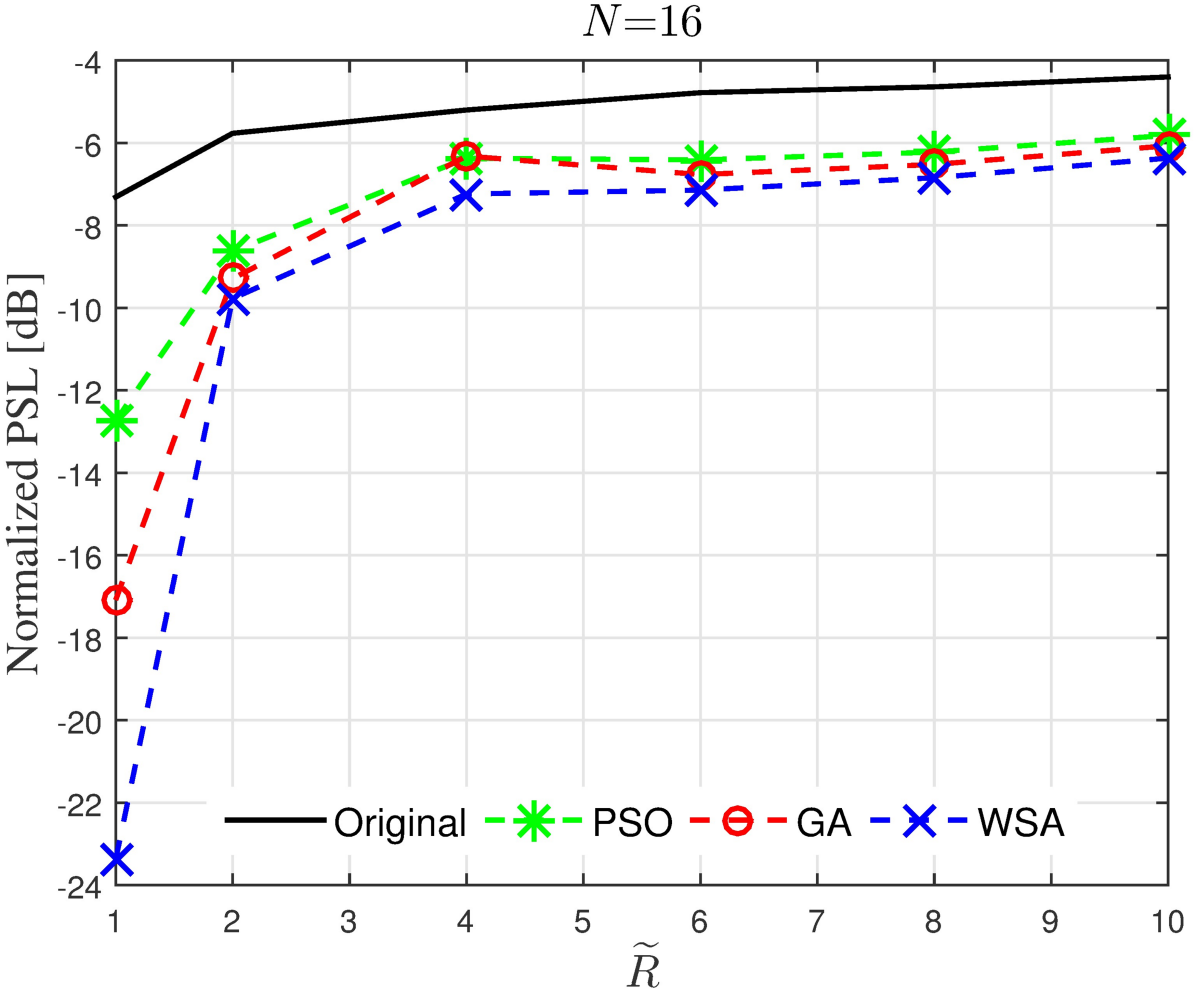
Comparisons of the PSL when $\tilde{R}$ is varied.

**Fig 10:**
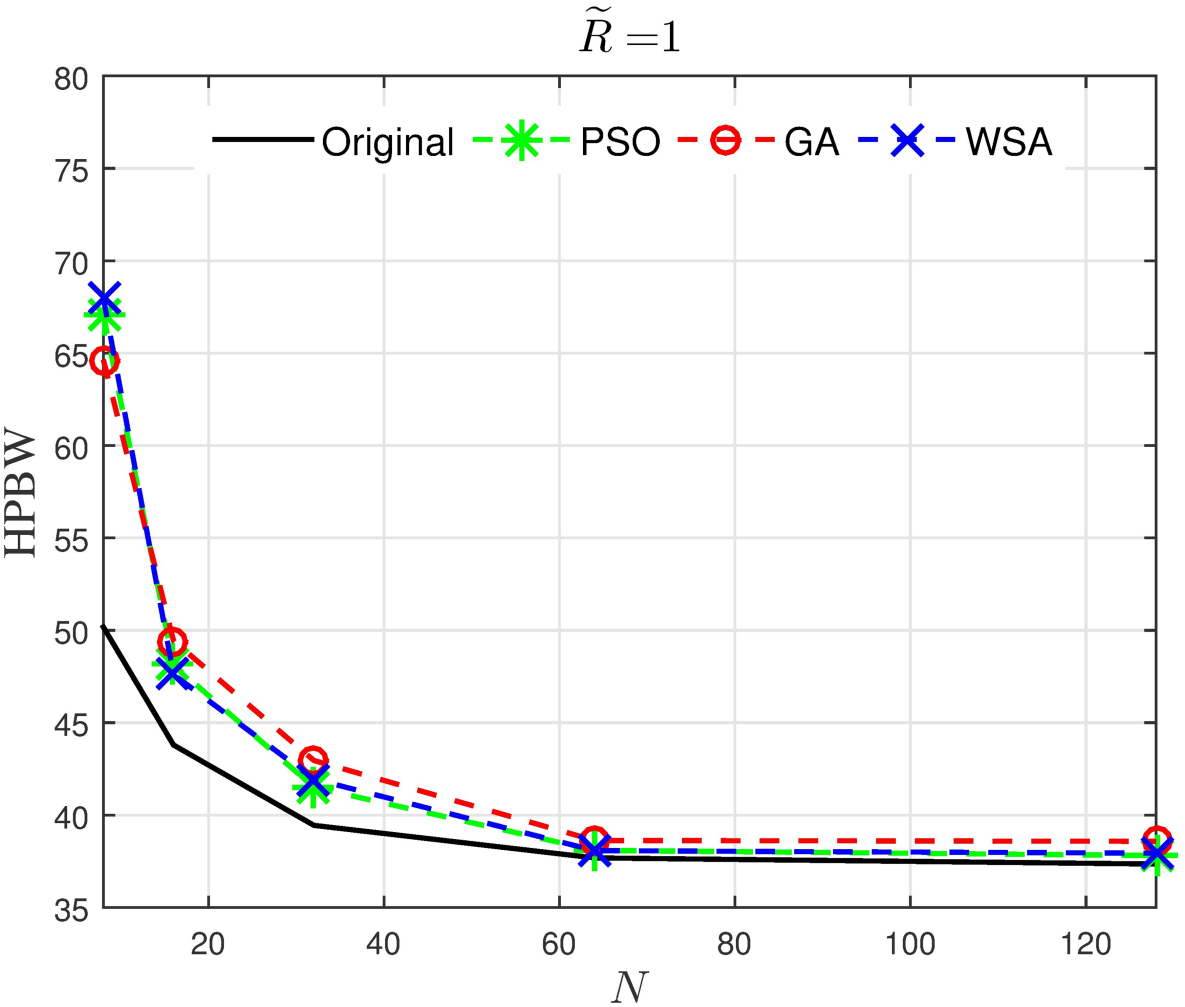
Comparisons of the HPBW when *N* is varied.

**Fig 11:**
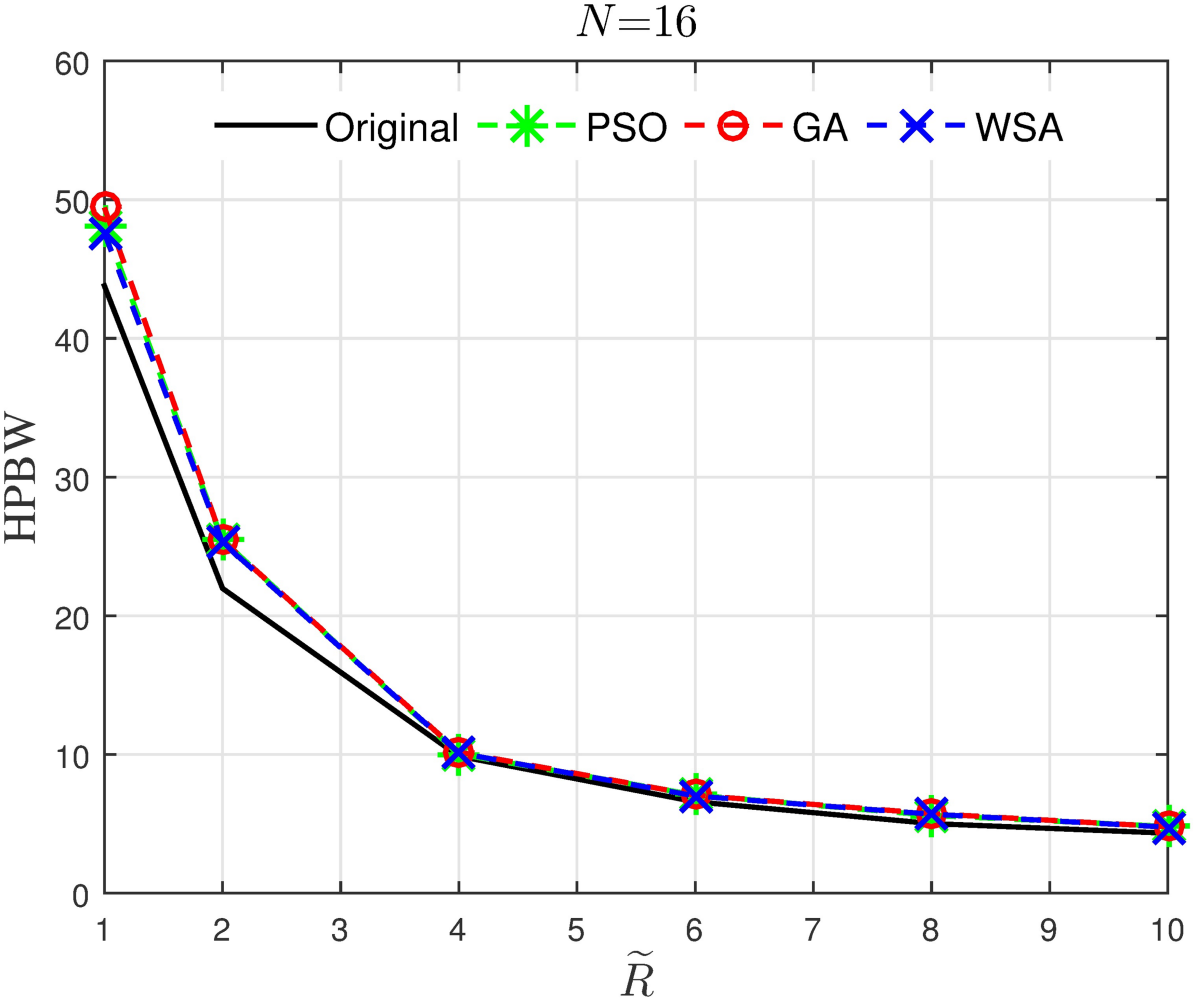
Comparisons of the HPBW when $\tilde{R}$ is varied.

Results in Figs 8–11 provide an insight on how the size of the cluster $\tilde{R}$ and the number of the collaborating nodes *N* affects the characteristics of the optimized beampattern. The average normalized peak sidelobe level and the average half-power beamwidth are recorded for a range of *N* and $\tilde{R}$. From Fig 8, it can be seen that amplitude optimization is consistently successful in reducing the PSL and is not affected by the number of nodes in the cluster. Out of the three optimization tools applied in this paper, the WSA provides the best improvement for the PSL reduction, where 15 to 20 dB improvement is recorded for the case of $\tilde{R}=1$ regardless of the number of collaborating nodes *N*. However, the improvement gradually decreases as the disk size of the cluster is increased. As can be seen in Fig 9, though all three optimization algorithms yield better average PSL compared to the conventional beamforming, the PSL improvement reduces from about 15 dB to 2 dB as the disk size increases from $\tilde{R}=1$ to $\tilde{R}=10$.

On the other hand, the optimization algorithms widen the HPBW of the beampattern, especially for a small number of collaborating nodes, as can be seen from Fig 10. While it is clear from the result that the HPBW reduces as the disk size $\tilde{R}$ increases as shown in Fig 11, the application of the optimization algorithms does not significantly alter the HPBW.

Hence, it can be concluded that when optimization algorithms are applied for the PSL reduction in the CBF, the improvement in the PSL reduction is not manipulated by *N*, but decreases when $\tilde{R}$ is increased. Meanwhile, the widening of the HPBW due to the optimization is not severely affected by $\tilde{R}$, and decreases as $\tilde{R}$ increases.

### 5.3 Capacity analysis

In this section, the impact of the sidelobes of a CBF on the capacity of an unintended receiver is investigated. To compare the capacity improvement, the rate of difference between the capacity of a proposed solution *C*_proposed_ a the benchmark solution of no optimization *C*_0_, where

$$\begin{array}{c} \begin{array}{c} \delta =\frac{C_{\text{proposed}}-C_{0}}{C_{0}}\times 100\% \end{array} \end{array}$$ (17)

A positive value for *δ* indicates an improved capacity performance. When the value of *δ* is negative, the proposed solution is performing worse than the benchmark CBF.

**Fig 12:**
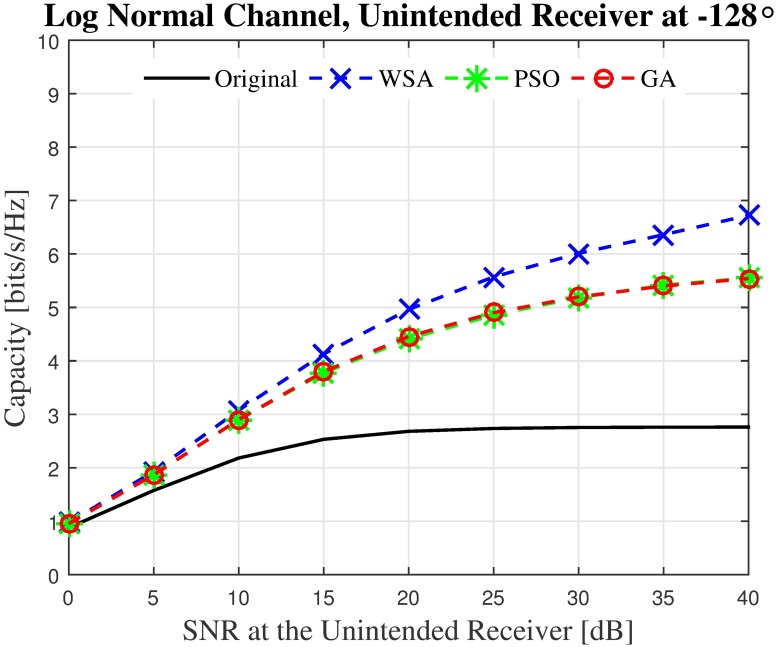
The instantaneous capacity at the unintended receivers in Fig 6 for all methods for unintended receiver at −128°.

**Fig 13:**
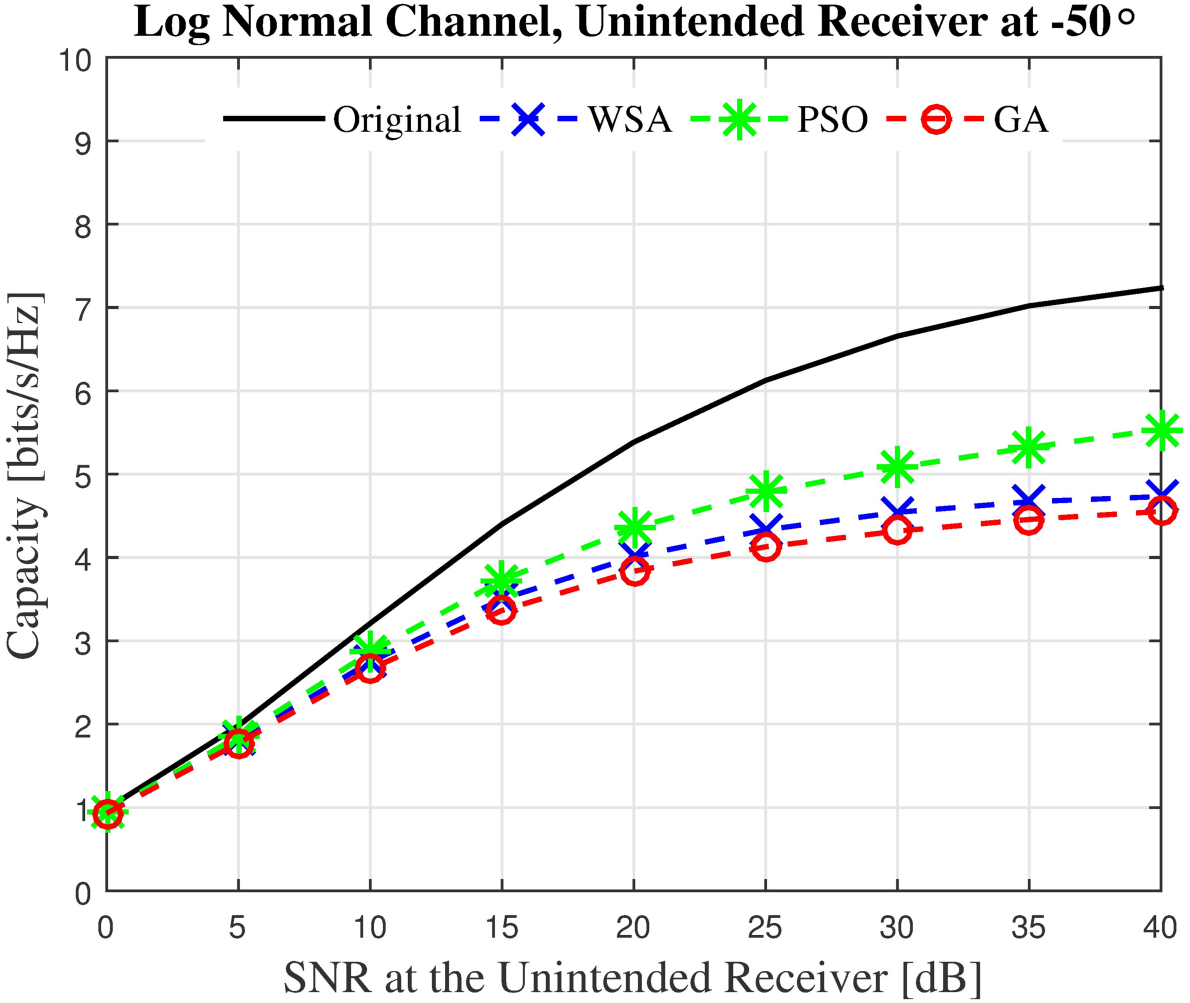
The instantaneous capacity at the unintended receivers in Fig 6 for all methods for unintended receiver at −50°.

**Fig 14:**
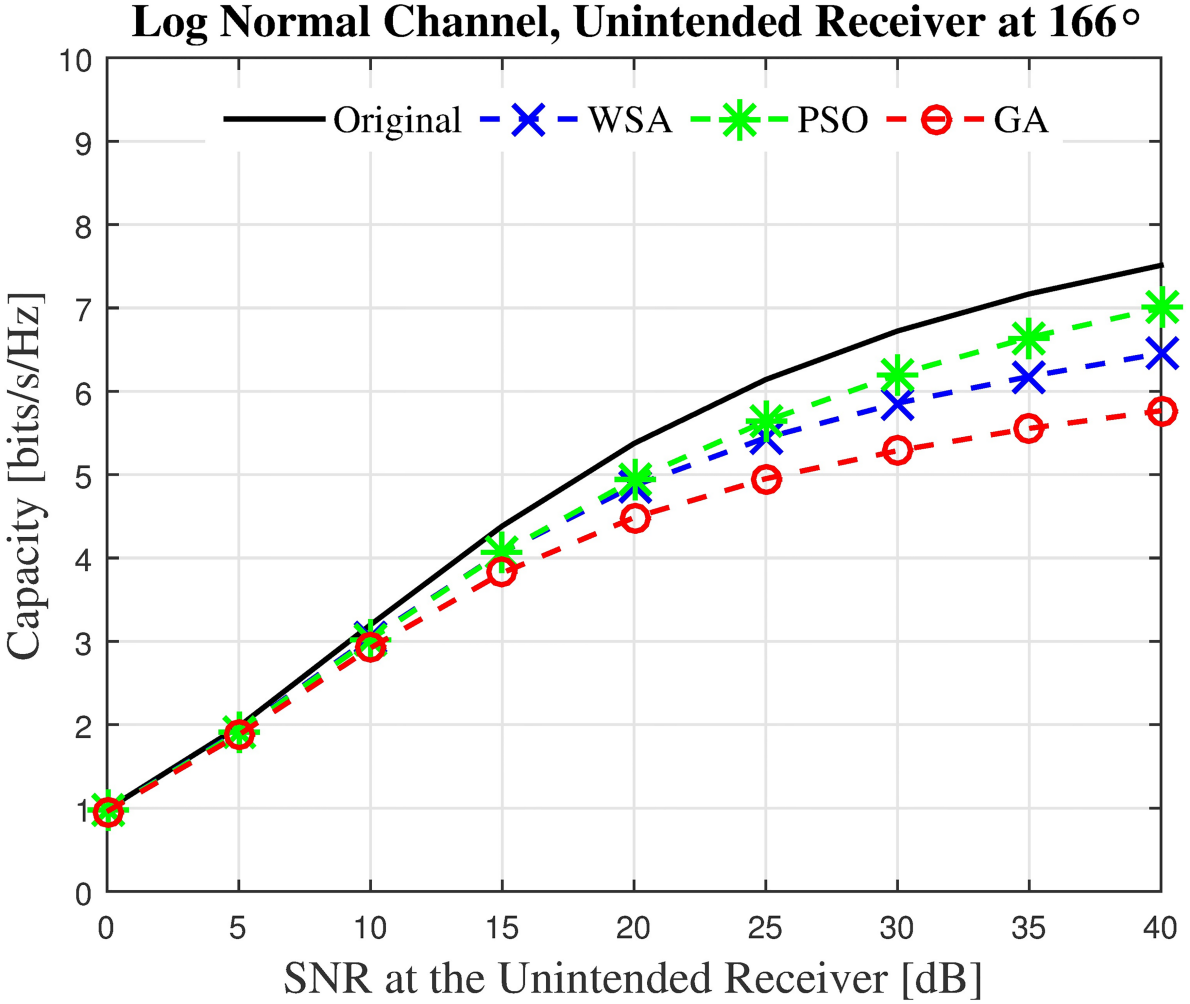
The instantaneous capacity at the unintended receivers in Fig 6 for all methods for unintended receiver at 166°.

**Fig 15:**
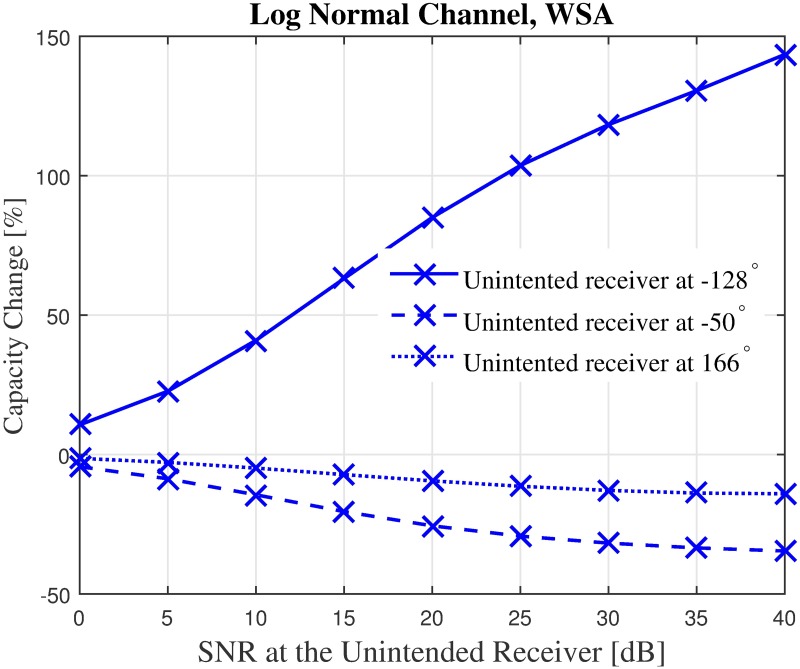
Percentage of the change for the instantaneous capacity at the unintended receivers in Fig 6 for WSA for unintended receivers at −128°, −50° and 166°.

First, the capacities of three unintended receivers located at the three critical points labeled in the sample beampattern in Fig 6 are analyzed. The capacity of the unintended receivers with the SNR values from 0 dB to 40 dB when disrupted by the CBF beampattern is investigated. For the first unintended receiver *U*_1_ which is located at the position of the PSL of the non-optimized beampattern, results in Fig 12 show that the WSA optimized beampattern could improve the capacity at the unintended receiver from 2.69 to 6.04 bits/Hz/s when the SNR is 40 dB, which accounts to 149% of improvement. However, when the unintended receiver is close to the mainbeam, the increased beamwidth of the optimized beampattern causes the capacity of the receiver located at that point *U*_2_ to reduce. The WSA beampattern optimization reduces the capacity at the unintended receiver *U*_2_ by −42.25% when the receive SNR is 40 dB, as shown in Fig 13. Similarly, Fig 14 shows that the capacity of the unintended receiver *U*_3_ located at 167°, records a negative change in capacity of −18.08% for the WSA due to the slight increase of the transmit power in this particular direction. The percentage of the change in capacity for all the three unintended receivers when their respective SNRs are [0, 40] dB for the WSA optimization are recorded in Fig 15.

Though there is a reduction in capacity (negative change in *δ*) at critical points *U*_2_ and *U*_3_ in the discussed sample beampattern, one has to keep in mind that in general, the transmit power obtained via optimization is lower at most locations, compared to the non-optimized beam pattern. Therefore, it can be deduced that applying the optimized weight for the CBF will improve the capacity at an unintended receiver located exactly at the location of the PSL (worst case) as well when located at a random location at most times.

**Fig 16:**
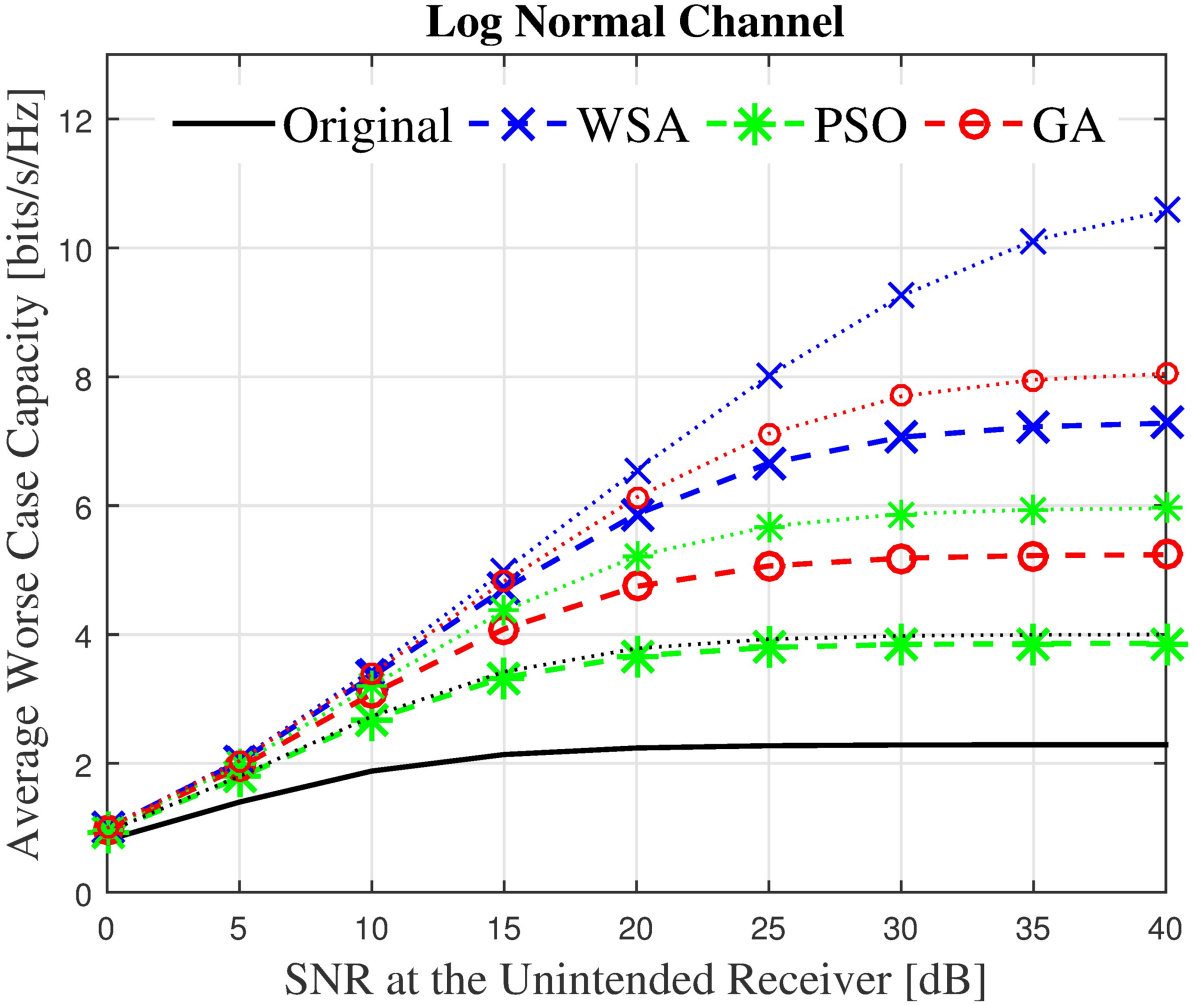
The average worst case capacity for *N* = 16 and *N* = 128.

**Fig 17:**
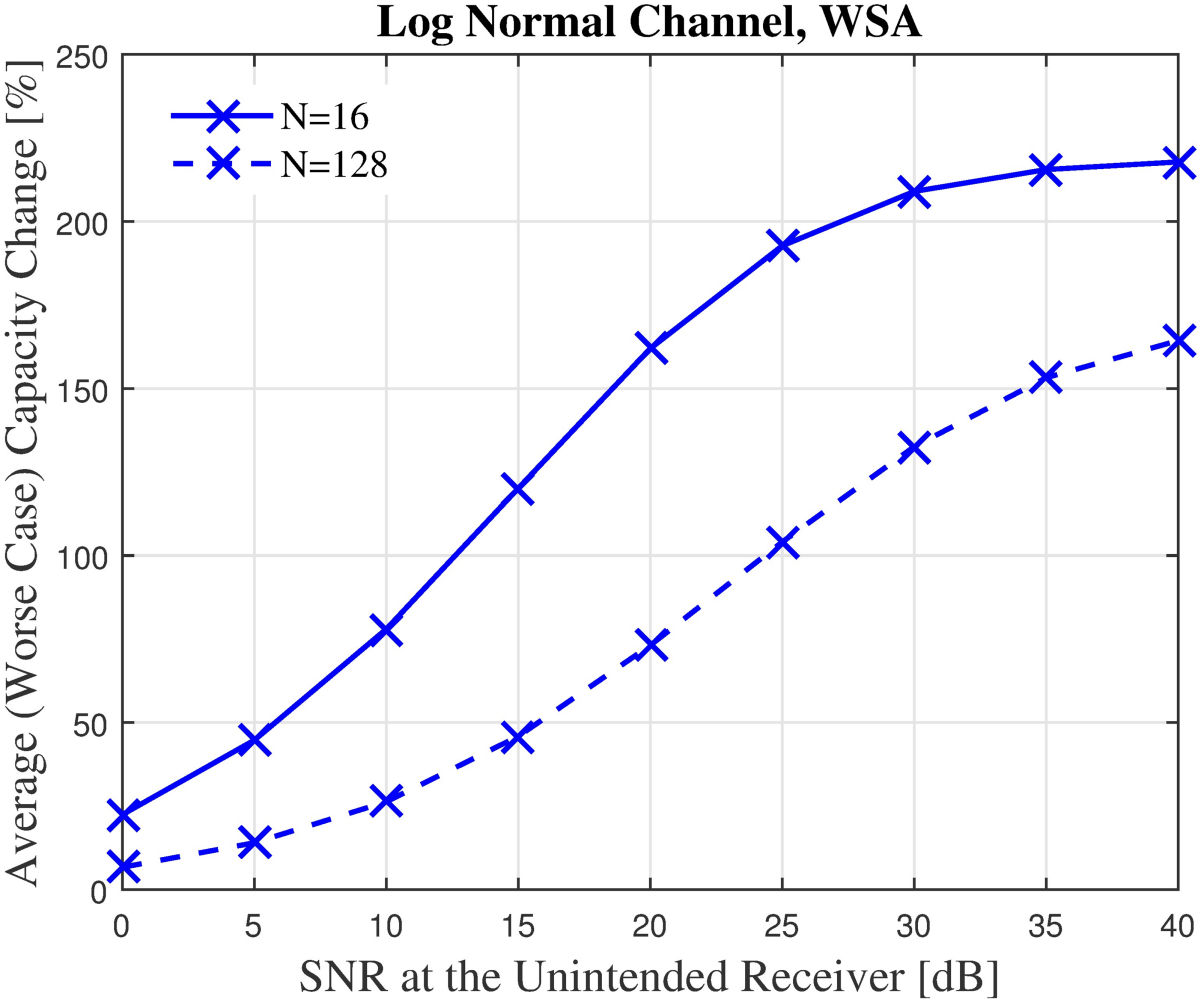
Percentage of the change in the WSA for *N* = 16 and *N* = 128.

Next, the capacity impact of an unintended receiver in a worst case scenario is analyzed, i.e., when the receiver is located exactly at the PSL of the CBF. An improvement in capacity is recorded for beampatterns optimized via all three optimization methods, as can be seen from Fig 16. The WSA optimized beampattern offers the best capacity improvement, and the *δ* of a WSA optimized beampattern is depicted in Fig 17. Up to 165% and 220% of capacity improvement is recorded when the number of collaborating nodes are *N* = 16 and *N* = 128, respectively.

**Fig 18:**
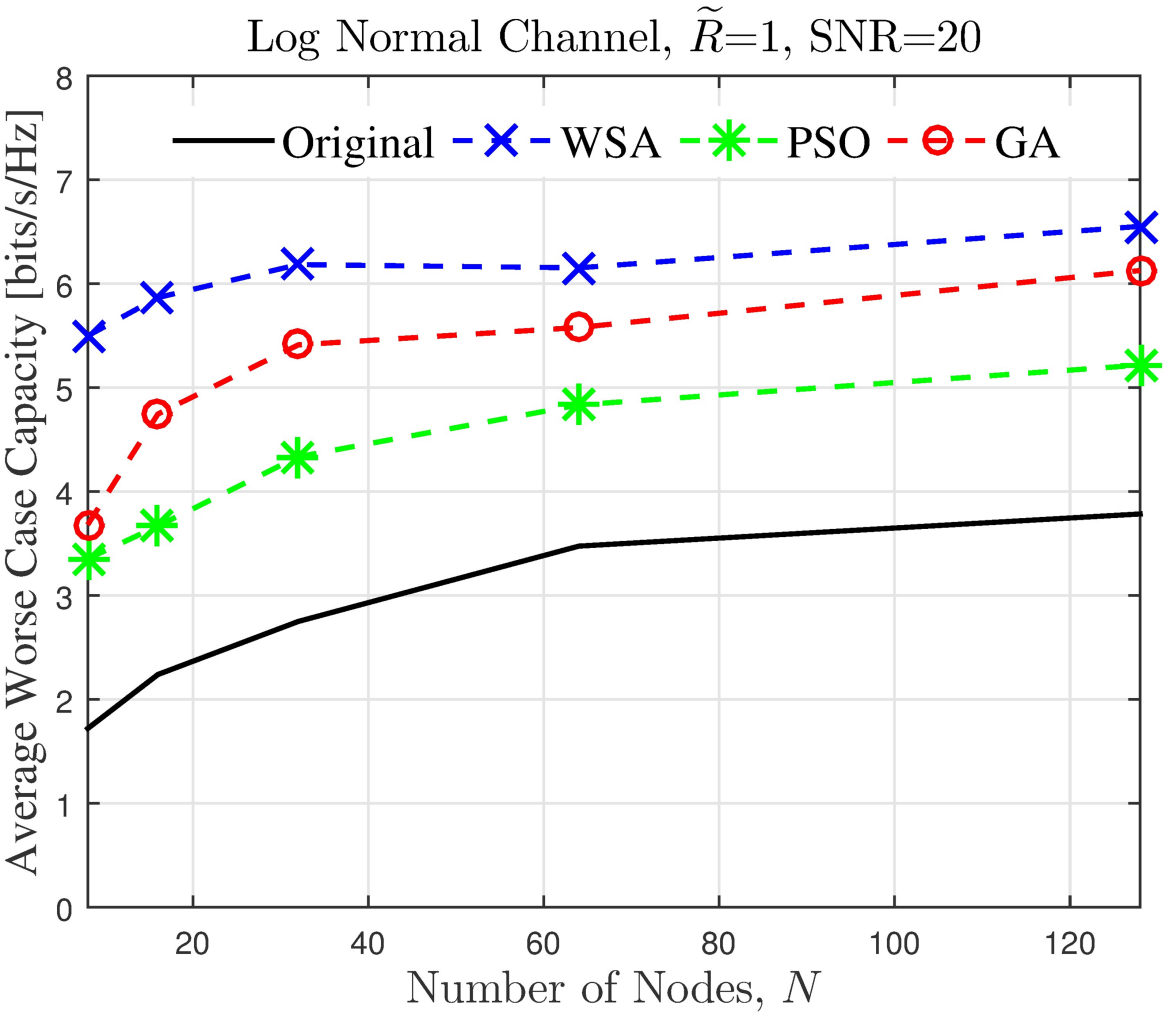
Comparisons on the worst case average capacity when number of collaborating nodes *N* is varied.

**Fig 19:**
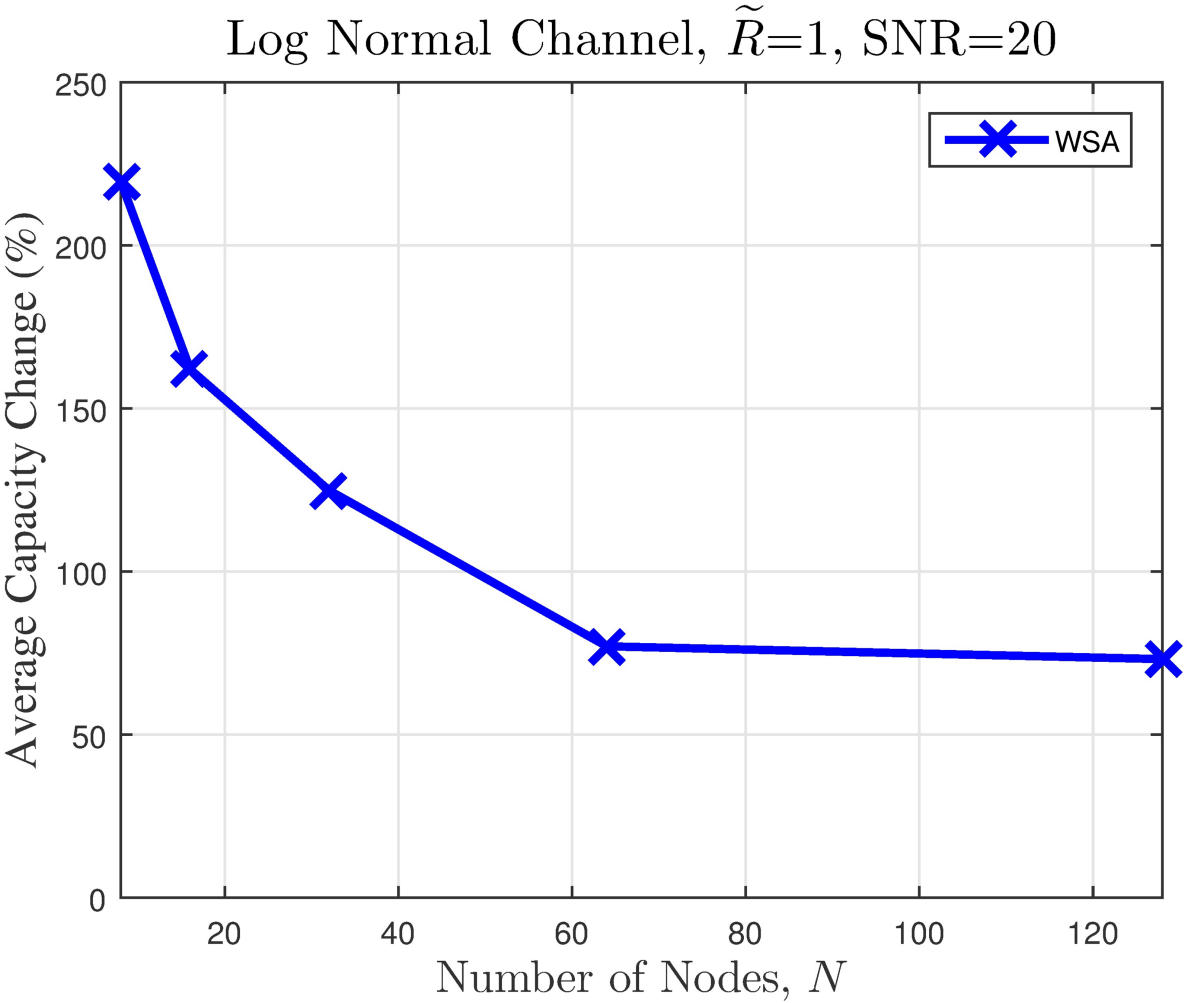
Comparisons on the worst case average percentage of the change in the WSA when number of collaborating nodes *N* is varied.

**Fig 20:**
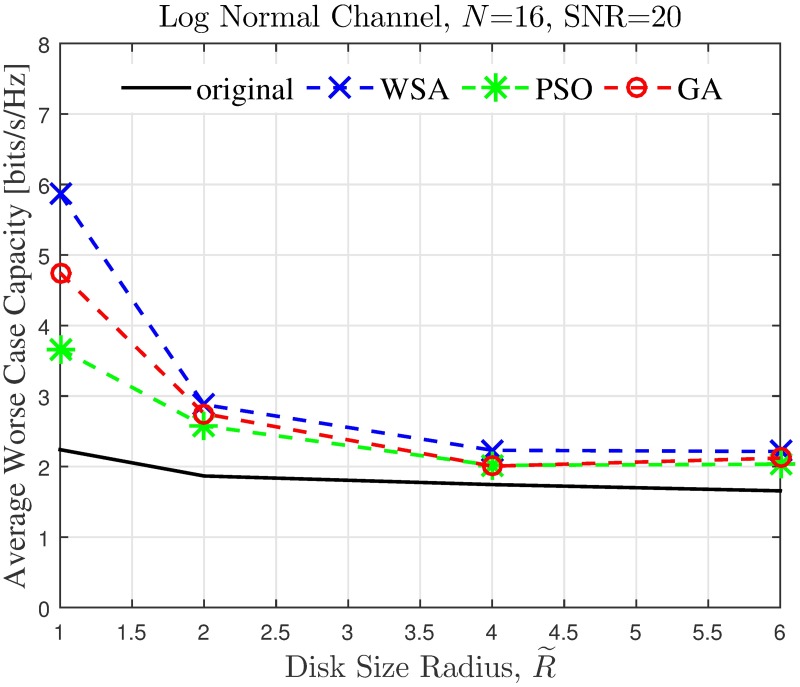
Comparisons on the worst case average improvement of the capacity when disk size $\tilde{R}$ is varied.

**Fig 21:**
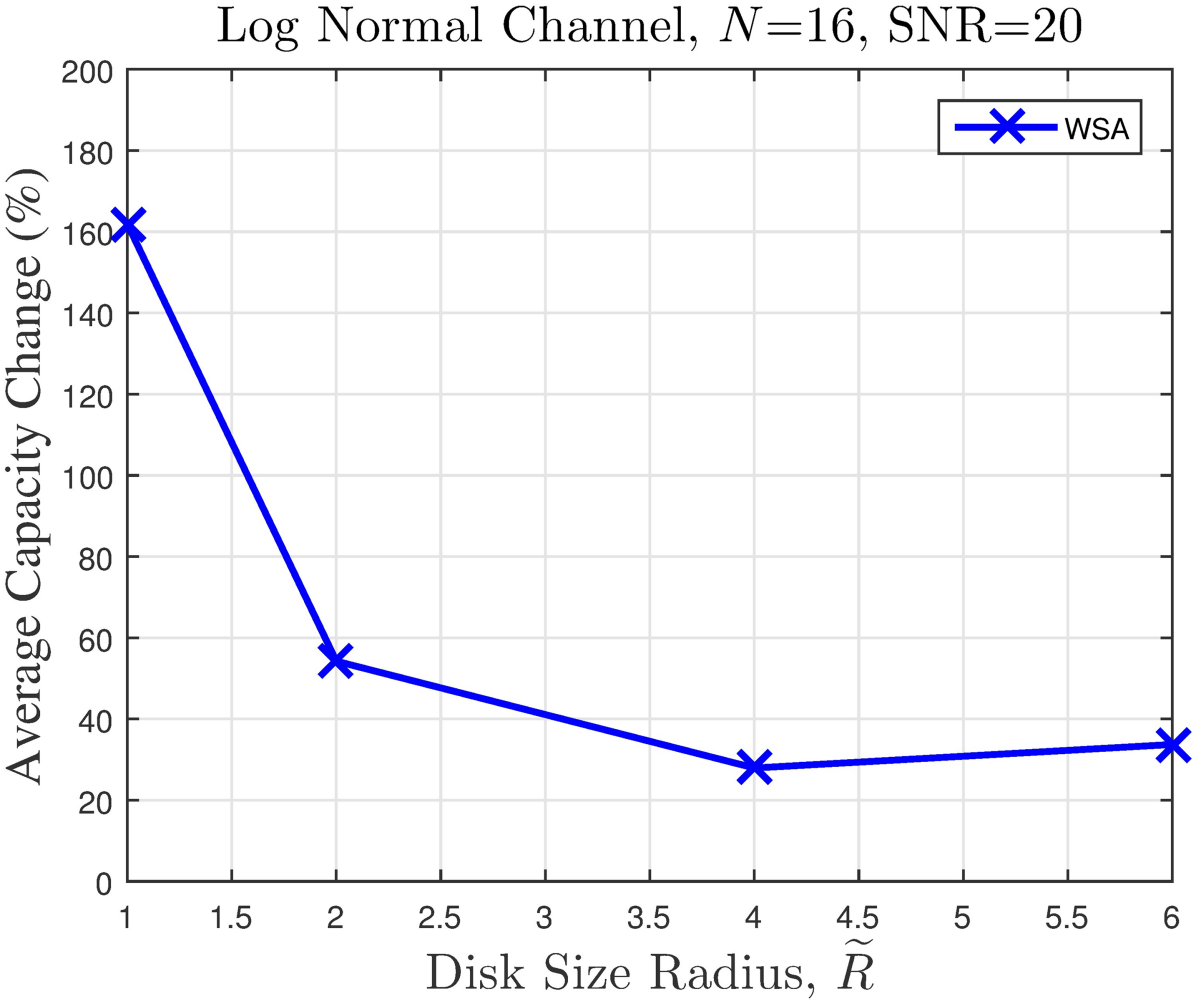
Comparisons on the worst case average percentage of the change in the WSA when disk size $\tilde{R}$ is varied.

It can be noted from Fig 16 that for the worst case scenario, the improvement increases as the number of nodes in the CBF cluster increases. To gain further insight on this phenomenon, the effects of the CBF cluster’s size and node density on the capacity of the unintended receiver in a worst case scenario is investigated for 250 samples and the average results are shown in Figs 18–21. It is interesting to note that, though it has been established from the beampattern analysis results in Fig 8 that the amount PSL reduction is fixed regardless of *N* for all beampattern optimization methods, lower number of nodes records higher capacity improvement, as illustrated in Figs 18–19. At lower *N*, the PSL is much higher and hence the interference level at the unintended receiver is much higher. Referring to Fig 3, reducing the interference when the interference level is high has more positive impact than when the interference level is low. This explains why the capacity improvement is higher for lower number of nodes in the CBF for the worst case scenario. Results in Figs 20–21 show that when the disk size of the CBF cluster increases, the capacity improvement at the unintended receiver due to the beampattern optimization decreases. This corroborates the beampattern analysis which has shown that the PSL minimization reduces as the disk size of the cluster increases.

**Fig 22:**
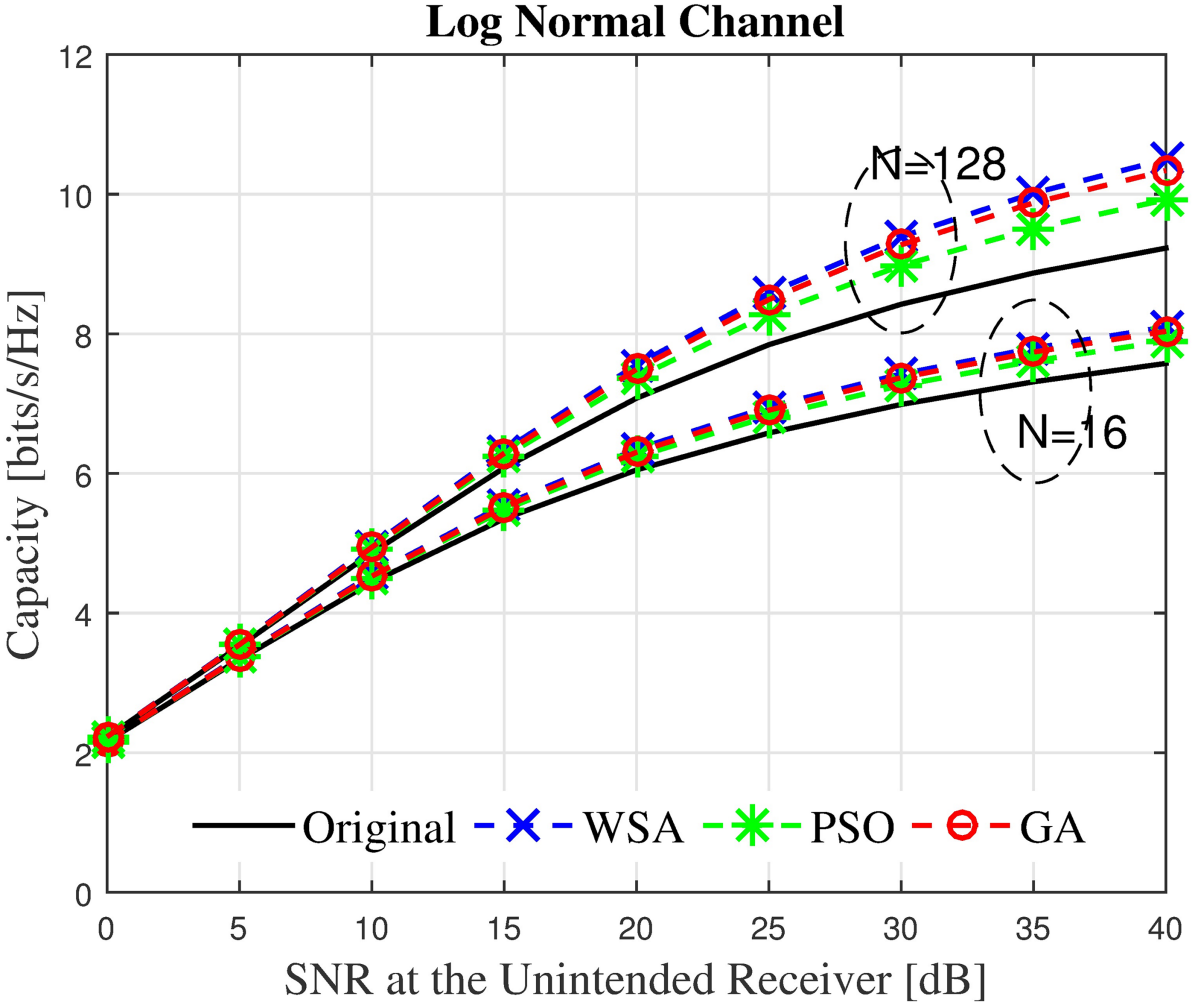
The average overall capacity for *N* = 16 and *N* = 128.

**Fig 23:**
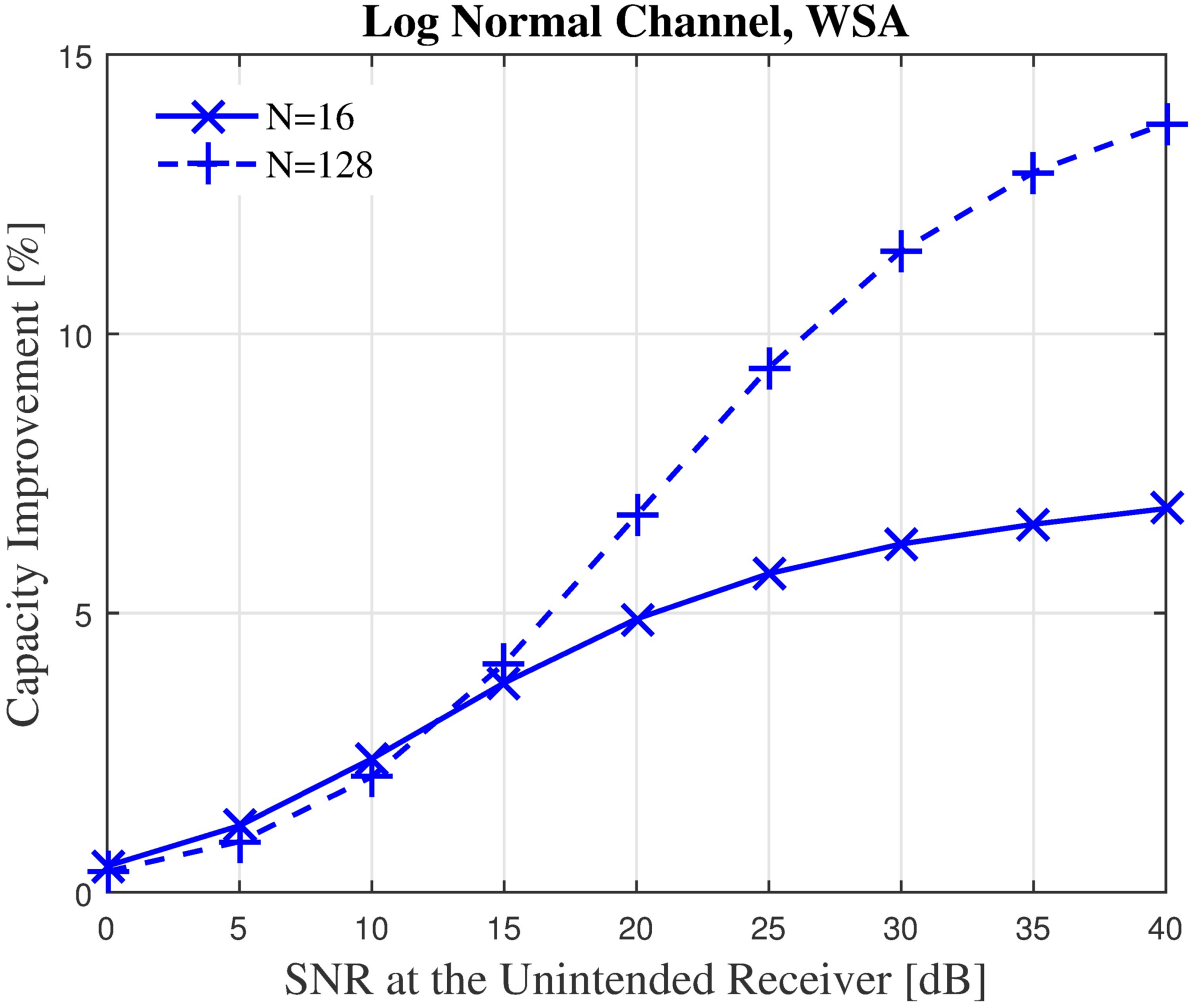
Percentage of the change for the WSA for *N* = 16 and *N* = 128.

Finally, to gain a more holistic overview on the effect of the beampattern optimization on an unintended receiver regardless of its location, the average capacity is analyzed by taking the mean capacity $\tilde{C}$ of 360 locations for the unintended receiver. The unintended receiver is randomly located at different positions, spread uniformly between *ϕ*_*U*_ ∈ [− 180°,180°] ≠ *ϕ*_0_. The average capacity of the unintended receiver with the SNR ranging between 0–40 dB in the presence of the interfering power from the CBF performed by a nearby cluster, with and without optimization is recorded in Figs 22–23 for *N* = 16, $\tilde{R}=1$ and *N* = 128, $\tilde{R}=1$. The capacity in the presence of shadowing at *N* = 16, $\tilde{R}=1$ improves from 7.5 bits/sec to 8 bits/ sec when the WSA based optimization is applied to the CBF. Similarly, for *N* = 128, $\tilde{R}=1$ the capacity at the unintended receiver improves from 9.2 bps to 10.3 bps at 40 dB SNR. A positive difference in capacity up to 13.7% and 6.87% is recorded for *N* = 128 and *N* = 16, respectively.

**Fig 24:**
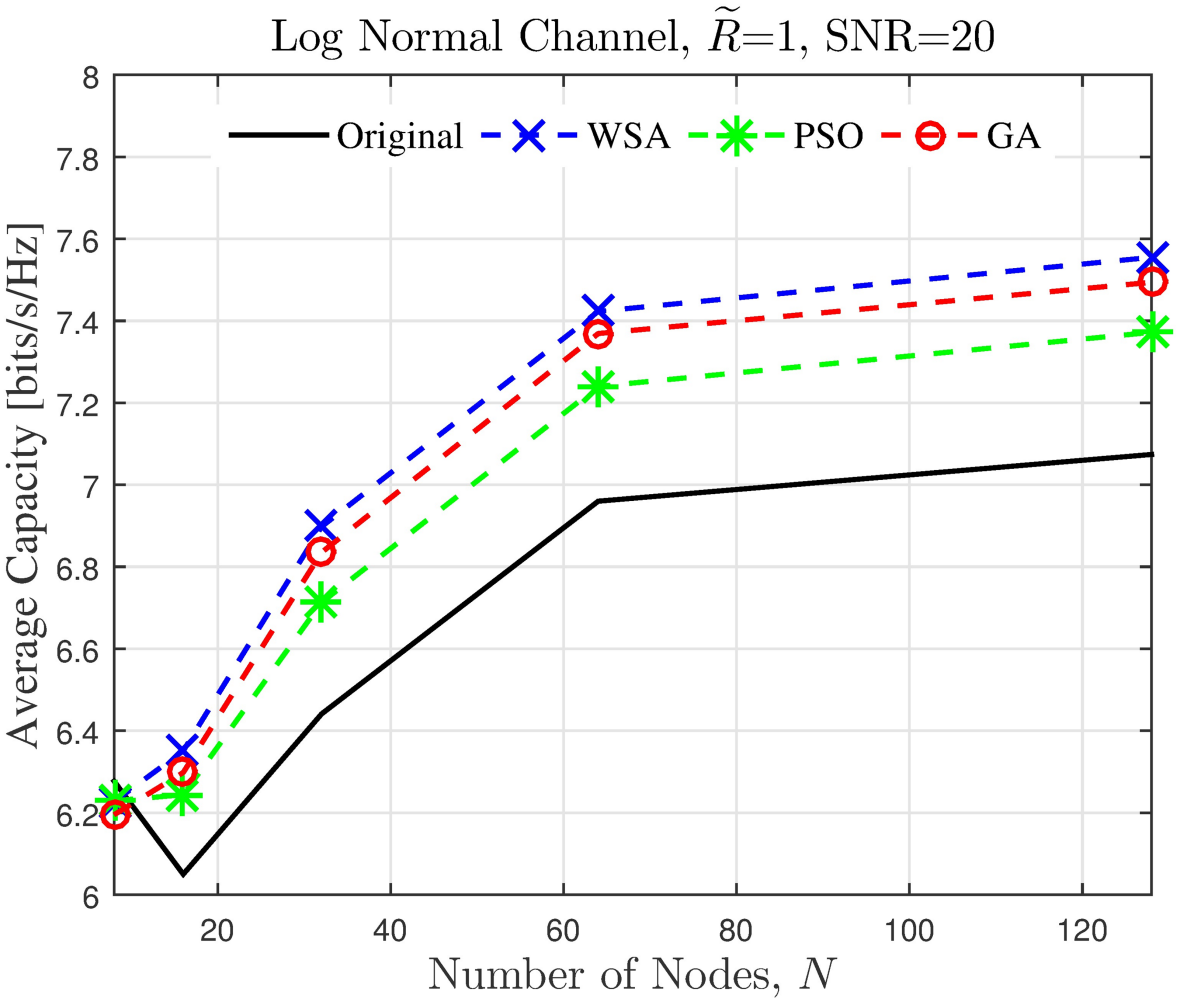
Comparisons on the overall average capacity when number of collaborating nodes *N* is varied.

**Fig 25:**
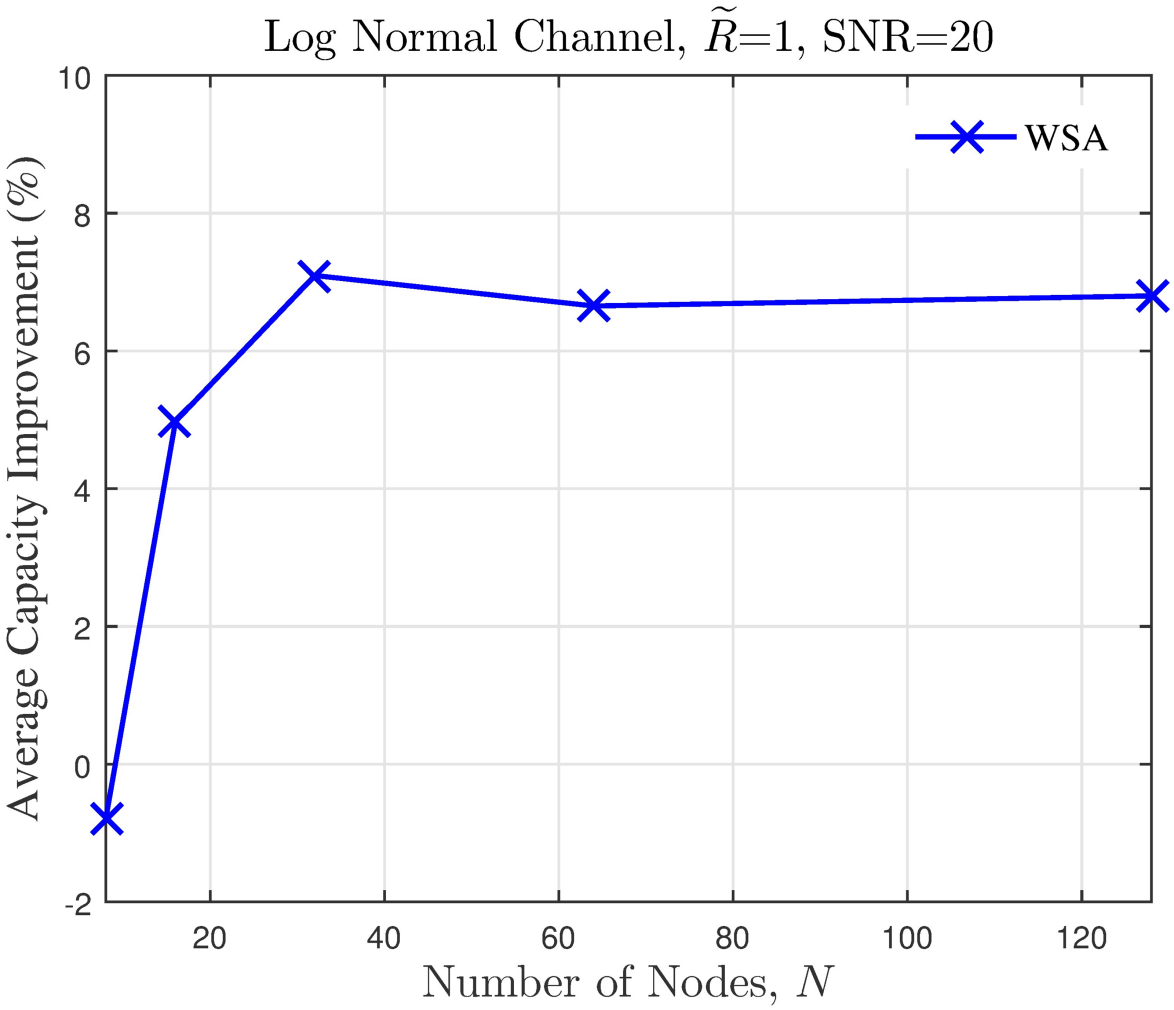
Percentage of the change in the WSA when number of collaborating nodes *N* is varied.

**Fig 26:**
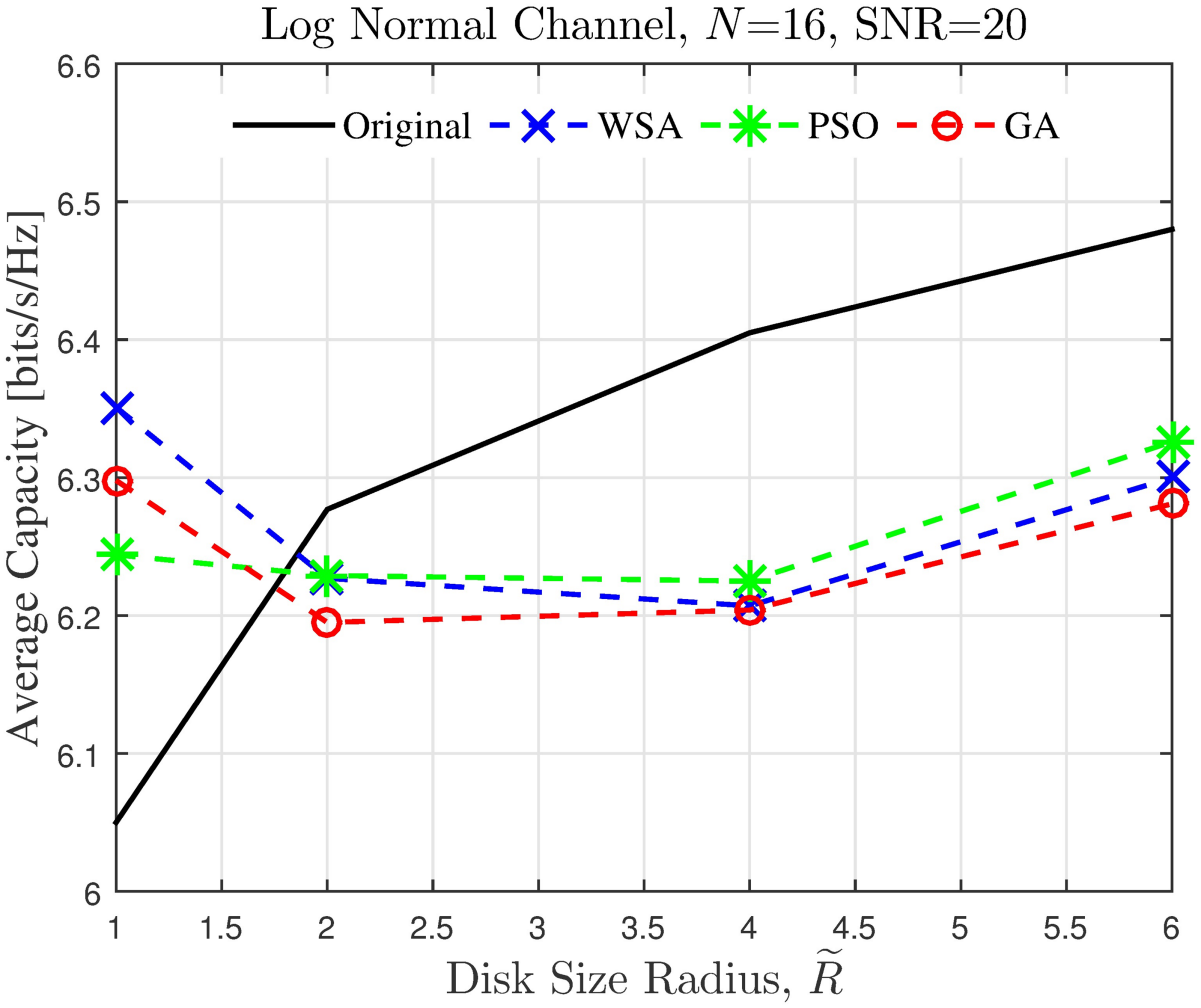
Comparisons on the overall average capacity when disk size of collaborating nodes $\tilde{R}$ is varied.

**Fig 27:**
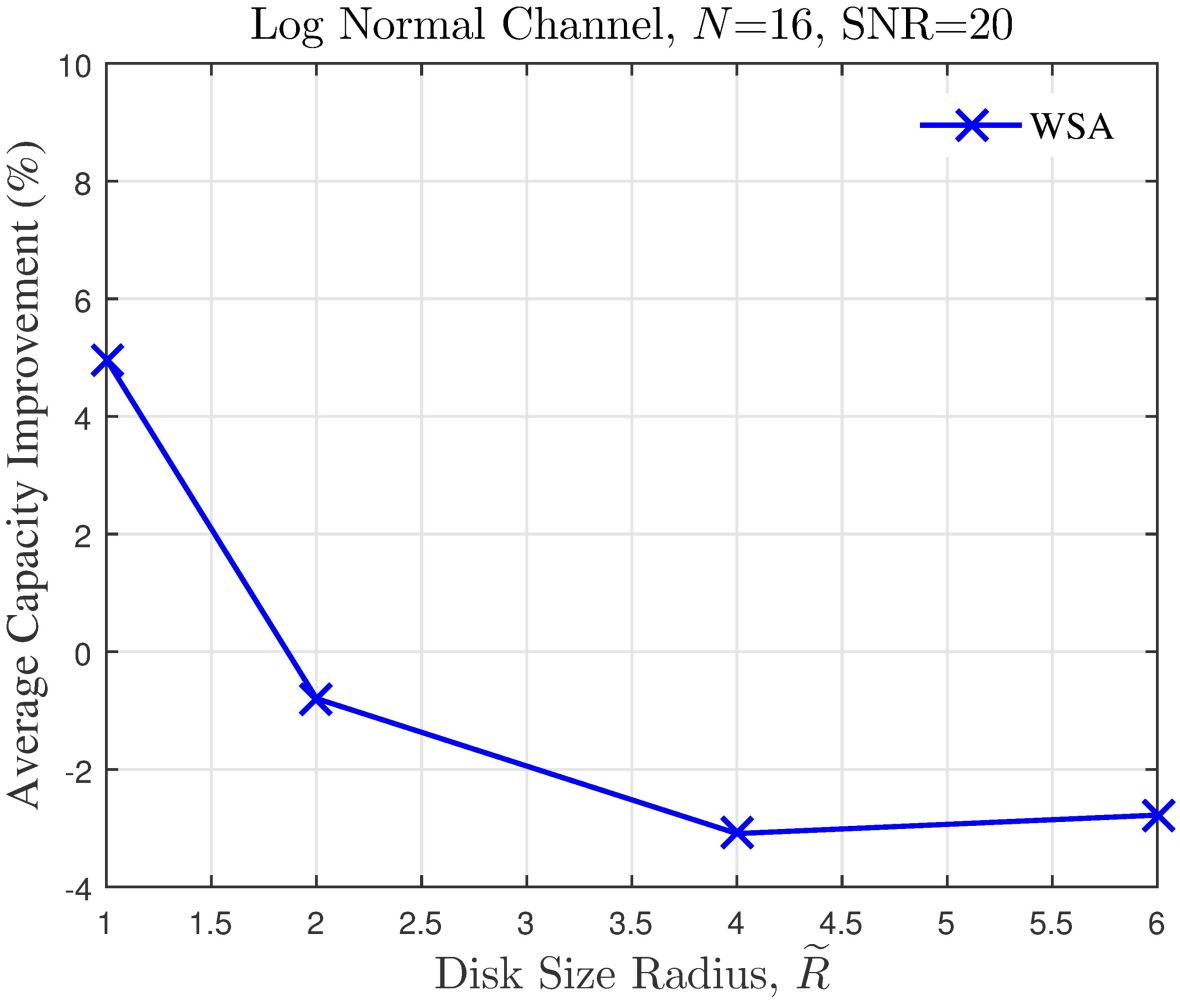
Percentage of the change in the WSA when disk size of collaborating nodes $\tilde{R}$ is varied.

The simulation is extended to study the effect of cluster dimension to the average capacity improvement of PSL optimized beampatterns in open-loop CBF. The results obtained in the presence of different dimensions of cluster sizes are presented in Figs 24–27 for a fixed SNR of 20 dB at the unintended receiver. Results show that for a fixed disk size, the optimization could provide an improvement in the capacity of the unintended receiver when the number of nodes is high. However, very limited improvement in capacity is recorded when the number of collaborating nodes is low. This can be attributed to the beampattern characteristics of beam formulated with a low number of nodes, which tends to have wider sidelobe. It has been established from the results in Fig 8 that the amplitude optimization to minimize the PSL provides the fixed reduction in the PSL regardless of the number of collaborating nodes *N*. However, optimization with lower *N* tends to widen the mainbeam, as discussed in Fig 10. Therefore, the probability of an unintended receiver falling within the mainbeam becomes higher and hence reduces the capacity of the receiver. Increasing the disk size of the cluster has detrimental effects on the capacity improvement as the improvement of PSL is lower for higher disk size $\tilde{R}$. By comparing the capacity difference in Fig 27 to the PSL improvements in Fig 9, it can be deduced a PSL reduction of at least 5 dB is needed to record a positive capacity difference at the unintended receiver.

The average capacity results provide valuable insight to how the optimization of PSL in the open-loop CBF affects unintended receivers located within its collaborative transmit range. Thus, it is observed that the optimization translates into improved capacity at the unintended receiver only when the disk size of the cluster is small. Higher capacity improvement is recorded when the number of collaborating nodes in the cluster is large. When the disk size of the cluster performing the CBF is large, the PSL optimization method is detrimental to the capacity of the unintended receiver. Therefore, PSL optimization in open-loop CBF is best suited for small clusters and high node density, for applications such as WSNs. In such application, it is best to utilize the proposed WSA as it provides the best capacity improvement at the unintended receivers.

## 6 Conclusion

PSL reduction in open-loop collaborative beamforming via amplitude perturbation and its effect on the capacity of unintended receivers are discussed in this paper. Optimization is performed using three meta-heuristic algorithms namely genetic algorithm (GA), particle swarm optimization (PSO) and weightless swarm algorithm (WSA). Results show that WSA suppresses the PSL better than the legacy PSO and the GA. Higher reduction in the PSL is achieved for the case of small clusters, and this translates to positive capacity improvement at an unintended receiver. The capacity analysis shows that the PSL optimization in open-loop collaborative beamforming is suited to clusters of small sizes and high node density, best for applications such as WSNs.

## Definitions of symbols

*A*: distance of the intended receiver relative to CH
*a*_RX_*k*__: fading gain between node *k* and an RX
*AF*: array factor of the CBF
*b*_RX_*k*__: path loss attenuation between node *k* and an RX
*β*: pointing error of the mainlobe
*C*: capacity at the unintended RX
*c*_1_: cognitive parameter in PSO and WSA
*c*_2_: social parameter in PSO
*f*: objective function of the optimization problem
*g*: constraint function of the optimization problem
*g*_best_: index of the global best solution in PSO and WSA
*h*_RX_*k*__: channel coefficient between node *k* and an RX
*I*: maximum number of iteration in the optimization process
*λ*: wavelength of transmitted signal
*M*: number of chromosome per generation in GA
*N*: number of collaborating nodes
*ω*: inertia weight in PSO and WSA
*P*_*k*_: transmission power of the signal at node *k*
*p*_best_: index of the local best solution in PSO and WSA
*ϕ*: azimuth angle of the intended receiver relative to CH
Ψ_*k*_: initial phase of the signal at node *k*
*ψ*_*k*_: azimuth angle of node *k* relative to CH
*R*: cluster radius
$\tilde{R}$
wavelength normalized cluster radius
*r*_*k*_: distance of node *k* relative to CH
*s*: transmission data
$\sigma_{a}^{2}$
variance of the fading distribution
$\sigma_{\text{n}}^{2}$
variance of the AWGN distribution
*r*_*k*_: distance of node *k* relative to CH
*ρ*: parent selection ratio in GA
*θ*: azimuth look angle of the CBF ranging from -*π* to *π*
*θ*_U_: azimuth angle of unintended RX relative to CH
w: additive white Gaussian noise at the receiver
*w*_*k*_: transmission weight of node *k* which is a function of *ξ*_*k*_ and *Ψ*_*k*_
*x*: position of a particle in PSO and WSA
*ξ*_*k*_: transmission amplitude of the signal at node *k*
*γ*: useful signal power at the unintended receiver
